# Prediction of depression treatment outcome from multimodal data: a CAN-BIND-1 report

**DOI:** 10.1017/S0033291722002124

**Published:** 2023-09

**Authors:** Mehri Sajjadian, Rudolf Uher, Keith Ho, Stefanie Hassel, Roumen Milev, Benicio N. Frey, Faranak Farzan, Pierre Blier, Jane A. Foster, Sagar V. Parikh, Daniel J. Müller, Susan Rotzinger, Claudio N. Soares, Gustavo Turecki, Valerie H. Taylor, Raymond W. Lam, Stephen C. Strother, Sidney H. Kennedy

**Affiliations:** 1 Department of Psychiatry, Dalhousie University, Halifax, NS, Canada; 2 University Health Network, 399 Bathurst Street, Toronto, ON, M5T 2S8, Canada; 3 Unity Health Toronto, St. Michael's Hospital, 193 Yonge Street, 6th floor, Toronto, ON, M5B 1M4, Canada; 4 Department of Psychiatry and Mathison Centre for Mental Health Research and Education, Cumming School of Medicine, University of Calgary, 3330 Hospital Dr NW, Calgary, AB, T2N 4N1, Canada; 5 Hotchkiss Brain Institute, University of Calgary, Calgary, AB, Canada; 6 Departments of Psychiatry and Psychology, Queen's University, Providence Care Hospital, Kingston, ON, Canada; 7 Department of Psychiatry and Behavioural Neurosciences, McMaster University, Hamilton, ON, Canada; 8 Mood Disorders Program and Women's Health Concerns Clinic, St. Joseph's Healthcare Hamilton, Hamilton, ON, Canada; 9 eBrain Lab, School of Mechatronic Systems Engineering, Simon Fraser University, Surrey, BC, Canada; 10 The Royal's Institute of Mental Health Research, 1145 Carling Avenue, Ottawa, ON, K1Z 7K4, Canada; 11 Department of Cellular and Molecular Medicine, University of Ottawa, 451 Smyth Road, Ottawa, ON, K1H 8M5, Canada; 12 Department of Psychiatry, University of Ottawa, 1145 Carling Avenue, Ottawa, ON, K1Z 7K4, Canada; 13 Department of Psychiatry & Behavioural Neurosciences, St Joseph's Healthcare, Hamilton, ON, Canada; 14 Department of Psychiatry, University of Michigan, Ann Arbor, MI, USA; 15 Campbell Family Mental Health Research Institute, Center for Addiction and Mental Health, Toronto, ON, Canada; 16 Department of Psychiatry, University of Toronto, Toronto, ON, Canada; 17 Department of Psychiatry, St Michael's Hospital, University of Toronto, Toronto, ON, Canada; 18 Department of Psychiatry, Queen's University School of Medicine, Kingston, ON, Canada; 19 Department of Psychiatry, Douglas Institute, McGill University, Montreal, QC, Canada; 20 Department of Psychiatry, Foothills Medical Centre, University of Calgary, Calgary, AB, Canada; 21 Department of Psychiatry, University of British Columbia, Vancouver, BC, Canada; 22 Rotman Research Center, Baycrest, Toronto, Canada; 23 Department of Medical Biophysics, University of Toronto, Toronto, Canada; 24 Department of Psychiatry, University Health Network, Toronto, Ontario, Canada; 25 Krembil Research Centre, University Health Network, University of Toronto, Toronto, Canada

**Keywords:** Machine learning, MDD, predictive analysis, treatment outcome

## Abstract

**Background:**

Prediction of treatment outcomes is a key step in improving the treatment of major depressive disorder (MDD). The Canadian Biomarker Integration Network in Depression (CAN-BIND) aims to predict antidepressant treatment outcomes through analyses of clinical assessment, neuroimaging, and blood biomarkers.

**Methods:**

In the CAN-BIND-1 dataset of 192 adults with MDD and outcomes of treatment with escitalopram, we applied machine learning models in a nested cross-validation framework. Across 210 analyses, we examined combinations of predictive variables from three modalities, measured at baseline and after 2 weeks of treatment, and five machine learning methods with and without feature selection. To optimize the predictors-to-observations ratio, we followed a tiered approach with 134 and 1152 variables in tier 1 and tier 2 respectively.

**Results:**

A combination of baseline tier 1 clinical, neuroimaging, and molecular variables predicted response with a mean balanced accuracy of 0.57 (best model mean 0.62) compared to 0.54 (best model mean 0.61) in single modality models. Adding week 2 predictors improved the prediction of response to a mean balanced accuracy of 0.59 (best model mean 0.66). Adding tier 2 features did not improve prediction.

**Conclusions:**

A combination of clinical, neuroimaging, and molecular data improves the prediction of treatment outcomes over single modality measurement. The addition of measurements from the early stages of treatment adds precision. Present results are limited by lack of external validation. To achieve clinically meaningful prediction, the multimodal measurement should be scaled up to larger samples and the robustness of prediction tested in an external validation dataset.

## Introduction

Over 25 antidepressant drugs and other therapies are effective in the treatment of major depressive disorder (MDD). While there are only small differences in the efficacy of the various treatments averaged across large groups of individuals, the response to each treatment varies substantially from individual to individual. Only a minority of individuals with MDD experience an adequate benefit from the first treatment they receive, leading many to sequentially try multiple treatments and combinations (Al-Harbi, 2012). Each unsuccessful treatment trial lasts several months and is associated with the risk of side effects, frustration, and adverse outcomes, including suicide. At present, the initial selection of treatment is usually based on evidence for efficacy and tolerability averaged across groups of individuals. Improvement in the selection of treatment requires tools that can predict whether a given individual will respond to a specific treatment. If this approach can be applied before, or early in the course of a treatment trial, those with a low likelihood of adequate therapeutic response can be redirected to treatment options that are more likely to be beneficial (Simon & Perlis, 2010).

Our knowledge of factors that predict treatment outcomes in depression has increased over the past decade. Known predictive factors include demographic characteristics (Fournier et al., 2009), history of adverse experiences (Nanni, Uher, & Danese, 2012), comorbid anxiety (Fava et al., 2008), symptom dimensions (Uher et al., 2012a), cognitive performance (Williams et al., 2011), molecular biomarkers (Uher et al., 2014), as well as measures of brain structure (Colle et al., 2018) and function (McGrath et al., 2013). While some of these predictive factors have been replicated across datasets, none is sufficiently accurate, robust, or economical for routine clinical use at an individual level.

MDD is a heterogeneous condition influenced by many factors that vary across individuals and populations. Therefore, it is likely that a more accurate individualized prediction can be achieved by models that consider multiple factors. Multivariate predictions can be constructed from models developed in existing datasets, using machine learning tools (Vu et al., 2018). Several groups of investigators have applied machine learning to depression treatment datasets to establish classifiers that could predict treatment outcomes (Chekroud et al., 2016; Etkin et al., 2015; Iniesta et al., 2018, 2016; Maciukiewicz et al., 2018; Nie, Vairavan, Narayan, Ye, & Li, 2018; Perlis, 2013). These results suggest that it is possible to construct parsimonious predictive models that use combinations of selected features from a large number of measurements to predict treatment effects for individuals who were not in the training dataset. A systematic review of published models found that studies using adequate methodology reported predictions with modest accuracy (Sajjadian et al., 2021). However, most published studies that used adequate methodology were limited to predictors of single modality, primarily those resulting from clinical questionnaires and interviews. Two prior studies that combined clinical features and molecular genetic markers reported improved accuracy of prediction compared to clinical features alone (Iniesta et al., 2018; Taliaz et al., 2021). The added benefit of multimodal measurement remains to be replicated and extended to additional data modalities, such as neuroimaging. It is also unknown whether the improved prediction is a function of combining data across measurement modalities, or is a result of including a larger number of predictors.

The goal of the Canadian Biomarker Integration Network in Depression (CAN-BIND) is to enhance treatment response in MDD through the prediction of treatment outcomes and personalized treatment strategies (Kennedy et al., 2012; Lam et al., 2016). In the present paper, we leverage the CAN-BIND-1 dataset to test whether multimodal measurements, composed of clinical, molecular, and brain imaging biomarkers improve the prediction of depression treatment outcomes. By systematically examining each domain of measurement and varying the number of predictive features in a tiered approach, we aim to answer the question of whether multimodal measurement or an increased number of variables influence the accuracy of prediction.

## Methods

### The CAN-BIND-1 dataset

The CAN-BIND-1 study enrolled 211 adults with MDD, who were extensively assessed, offered treatment with the serotonin-reuptake inhibiting antidepressant escitalopram (10–20 mg), and invited for follow-up assessments every two weeks for 16 weeks (Kennedy et al., 2019; Lam et al., 2016). Of these, 192 (91%) attended the assessment after 2 weeks, 180 (85%) attended the assessment 8 weeks after treatment initiation, and 166 (79%) attended the final planned assessment after 16 weeks of treatment. At week 8, participants who did not respond to escitalopram were offered additional treatment with aripiprazole (2–10 mg) as an augmenting strategy. CAN-BIND-1 was approved by the Research Ethics Boards at all recruiting sites. All participants signed an informed consent after the study procedures had been explained. The present study uses data from the first 8 weeks when all participants received escitalopram as a monotherapy. We include 192 participants (74 men and 118 women, mean age 35.4, s.d. = 12.8 years) who provided valid outcome data on one or more follow-ups after initiating treatment (see online Supplementary Table S1 for a comparison of participants who did and who did not contribute to analyses). Analyses that included predictors from week 2 used a subset of 188 participants (71 men and 117 women, mean age 35.3, s.d. = 12.7 years) who provided valid outcomes at week 4 or later. Further details of the CAN-BIND-1 clinical dataset are available elsewhere (Kennedy et al., 2019; Lam et al., 2016). The detailed flow diagram of CAN-BIND-1 participants is depicted in online Supplementary Fig. S1.

### Outcomes of antidepressant treatment

In CAN-BIND-1, we measured the severity of depressive symptoms every 2 weeks for 16 weeks with the clinician-rated Montgomery and Åsberg Depression Rating Scale (MADRS). At baseline, the participants were moderately to severely depressed, scoring on average 30 on MADRS (range 21–47). The improvement in depressive symptoms with treatment can be indexed with a continuous measure (absolute or proportional reduction) or a dichotomized categorical outcome (remission, response). Consistent with previous studies in the field, we chose to use a categorical outcome to provide a metric that is comparable with prior literature (Sajjadian et al., 2021). Categorical outcome measures based on absolute numbers of the end-point score (remission) and proportion of change from baseline score (response) have complementary advantages and disadvantages. The probability of remission is negatively related to baseline severity, but the probability of response is independent of severity at baseline (Coley et al., 2020). Since we aimed to index improvement in a way that is independent of baseline severity (Kennedy et al., 2012), we chose response, defined as a reduction in MADRS by 50% or more from baseline to week 8, as the primary outcome measure. When MADRS at week 8 was missing, we used earlier time points to estimate the outcome based on mixed-effects models for repeated measures, as previously described (Uher et al., 2020). When no outcome data were available at any post-baseline time point (or post-week-2 for analyses that used week-2 measurements as predictors), we did not include the participant in any analyses. The outcome response rates were 46.9% and 47.3% for baseline and week 2 samples respectively.

### Predictors

At baseline (week 0), CAN-BIND-1 participants underwent detailed assessments with interviews, questionnaires, cognitive testing, magnetic resonance neuroimaging, and blood sampling. The assessments were organized into three modalities: (1) *Clinical modality* included interviews to establish diagnoses, medical history, current severity of depression, and functioning, questionnaires covering depressive and anxiety symptoms, personality traits, and functioning, cognitive testing, and measurement of body weight and height to calculate body mass index (Kennedy et al., 2019; Lam et al., 2016). (2) *Molecular modality* used blood samples to extract DNA for genomic and epigenetic analyses, and measure a comprehensive panel of micro RNAs, levels of common metabolites, and inflammatory markers. (3) *Neuroimaging modality* used multimodal magnetic resonance imaging to obtain whole-brain structural T1 and T2-weighted images, diffusion tensor imaging of the white matter, and functional magnetic resonance imaging during resting-state and depression-relevant tasks (Macqueen et al., 2019). A subset of measurements was repeated after 2 weeks (week 2). Further details of CAN-BIND-1 assessments are available elsewhere (Kennedy et al., 2019; Lam et al., 2016).

### Tiered selection of predictors for analysis

To optimize the use of the rich dataset with a limited sample size, we adopted a two-tiered approach to the inclusion of potential markers in the predictive model development with tiers 1 and 2 considering focused and comprehensive sets of potential predictors, respectively. *Tier 1* predictors were selected based on prior published evidence of predictive value, measurement reliability, data completeness, and conceptual value which were pre-processed, derived, or engineered (e.g. total scale score was used rather than individual questionnaire items, selected regional brain volumes rather than voxel-level signal intensity, total DNA methylation rather than methylation at specific genomic loci). The exact number of tier 1 predictors was not determined *a priori*; however, the aim was to retain several predictors that are similar to or lower than the number of individuals in the analytic sample. The selection process resulted in a set of 134 variables measured at baseline that represented all three measurement modalities in tier 1 analyses (Table 1). For analyses including baseline and week 2 predictors, an additional 80 variables from week 2 assessments were added to the baseline tier 1 variables, resulting in a total of 214 predictors (Table 1). Where prior evidence was used in the selection of predictors, it was strictly limited to prior studies that did not use CAN-BIND-1 participants. Tier 2 predictors were also mostly pre-processed, and adequately measured, but were included without any requirement of prior evidence of predictive value. The inclusion of a greater number of predictors allowed considering more comprehensive and granular information (e.g. sub scores or items from a questionnaire, volume measurements of all brain regions, all known micro RNAs measured with adequate reliability). In tier 2 analysis, we included 1152 predictor variables measured at baseline that comprehensively cover the key information from the three assessment modalities (Table 1). The gradual inclusion of predictors by tier and by modality allowed us to separate the contribution of multimodal measurement from the effect of a greater number of predictors, examine the limits of machine learning analyses in a moderately sized clinical sample and test the value of prior evidence in variable selection. The list and number of predictors considered for inclusion in each tier are given in online Supplementary Table S2. A description of all tier 1 variables is given in online Supplementary Table S3.

**Table 1.** Predictive models achieving the highest mean balanced accuracy in tier 1 and tier 2 dataset

Analysis	Modality	Predictive week	Machine learning method	Feature selection	# of variables before feature selection	# of variables after feature selection	Sensitivity	Specificity	PPV	NPV	Balanced accuracy	AUC
Tier 1	Clinical + neuroimaging + molecular	Week 0	SVM	None	134	134	0.45	0.78	0.65	0.62	0.62	0.64
Tier 1	Clinical	Week 0	Naïve Bayes	CAT score	47	25	0.56	0.60	0.55	0.61	0.58	0.58
Tier 1	Molecular	Week 0	SVM	None	31	31	0.49	0.73	0.62	0.62	0.61	0.61
Tier 1	Neuroimaging	Week 0	Naïve Bayes	None	56	56	0.53	0.61	0.55	0.60	0.57	0.58
Tier 1	Clinical + neuroimaging	Week 0	Naïve Bayes	None	103	103	0.57	0.63	0.58	0.62	0.60	0.60
Tier 1	Neuroimaging + molecular	Week 0	SVM	CAT score	87	25	0.50	0.66	0.57	0.60	0.58	0.60
Tier 1	Clinical + molecular	Week 0	SVM	None	78	78	0.47	0.71	0.59	0.60	0.59	0.63
Tier 2	Clinical + neuroimaging + molecular	Week 0	SVM	CAT score	1152	25	0.52	0.60	0.54	0.59	0.56	0.59
Tier 2	Clinical	Week 0	Random forest	Embedded + CAT score	194	25	0.51	0.62	0.54	0.59	0.57	0.59
Tier 2	Molecular	Week 0	Elastic net	Embedded	733	733	0.28	0.77	0.51	0.55	0.52	0.54
Tier 2	Neuroimaging	Week 0	GBM	Embedded	225	225	0.49	0.60	0.52	0.57	0.55	0.55
Tier 2	Clinical + neuroimaging	Week 0	Random forest	CAT score	419	25	0.51	0.64	0.56	0.60	0.58	0.59
Tier 2	Neuroimaging + molecular	Week 0	SVM	CAT score	958	25	0.49	0.61	0.52	0.58	0.55	0.58
Tier 2	Clinical + molecular	Week 0	Naïve Bayes	CAT score	927	25	0.63	0.49	0.53	0.60	0.56	0.59
Tier 1	Clinical + neuroimaging + molecular	Week 0 + week 2	Random forest	Embedded	214	214	0.59	0.73	0.67	0.67	0.66	0.71
Tier 1	Clinical	Week 0 + week 2	Elastic net	Embedded	55	55	0.52	0.80	0.71	0.65	0.66	0.71
Tier 1	Molecular	Week 0 + week 2	SVM	None	52	52	0.35	0.84	0.67	0.61	0.60	0.62
Tier 1	Neuroimaging	Week 0 + week 2	Naïve Bayes	None	107	107	0.57	0.59	0.55	0.60	0.58	0.58
Tier 1	Clinical + neuroimaging	Week 0 + week 2	Random forest	Embedded	162	162	0.59	0.73	0.67	0.67	0.66	0.70
Tier 1	Neuroimaging + molecular	Week 0 + week 2	Naïve Bayes	None	159	159	0.65	0.48	0.53	0.62	0.57	0.61
Tier 1	Clinical + molecular	Week 0 + week 2	Elastic net	Embedded	107	107	0.51	0.81	0.71	0.65	0.66	0.73

### Missing values

Missing values are inevitable in human datasets and the way missing values are handled can influence the results. One commonly used approach is to only include individuals with valid values for all variables, referred to as complete case analysis which introduces a bias in all cases other than when all values are missing completely at random. The preferred alternative to complete case analysis is the imputation of missing values. For imputation of missing values on *predictor* variables, we chose ‘missRanger’ due to its capability to handle non-normal distributed data with various types of predictors (Stekhoven & Bühlmann, 2012). ‘MissRanger’ was introduced by Mayer et al. for the imputation of multimodal datasets, similar to CAN-BIND-1 dataset (Stekhoven & Bühlmann, 2012). It uses a fast implementation of random forest package ‘ranger’ (Wright & Ziegler, 2017) and includes predictive mean matching which prevents imputation with values that do not exist in the original data such as a value 0.5 in a 0–1 binary variable. Importantly, missRanger was integrated into the machine learning workflow so that imputation was done independently in each training and each testing set, preventing information leakage. Outcome measures were not included in the imputation procedure (see section *Outcomes of Antidepressant Treatment*, above, for missing values on outcomes). For visualization of missing values patterns, please see online Supplementary Fig. S2*A*–*I*.

### Development and assessment of prediction models

**Fig. 1.**
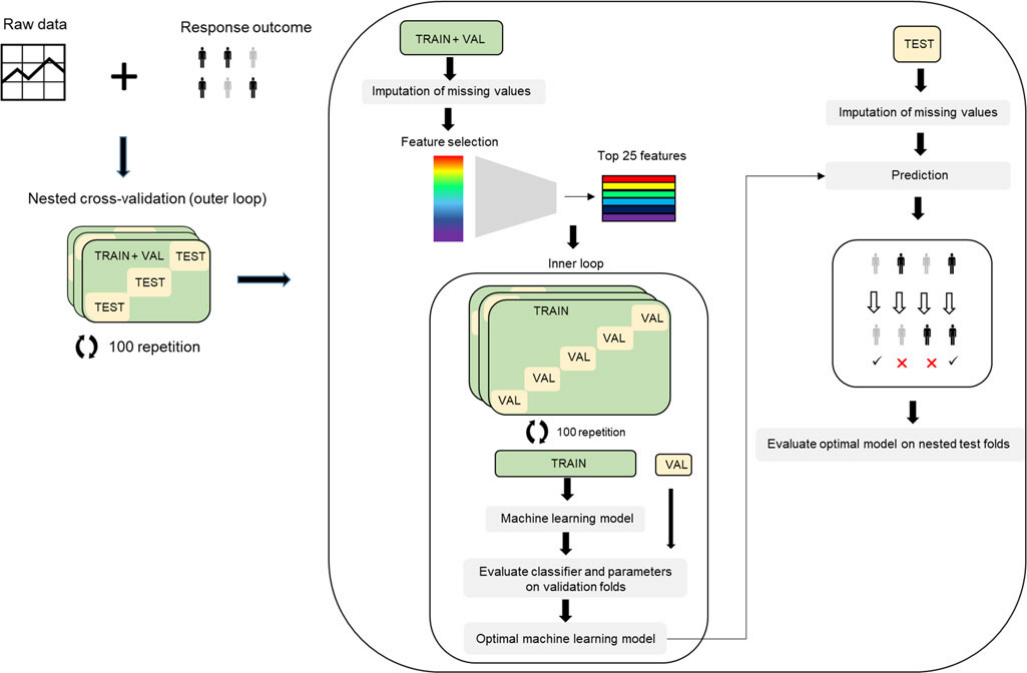
Analysis workflow of treatment outcome prediction model.

**Fig. 2.**
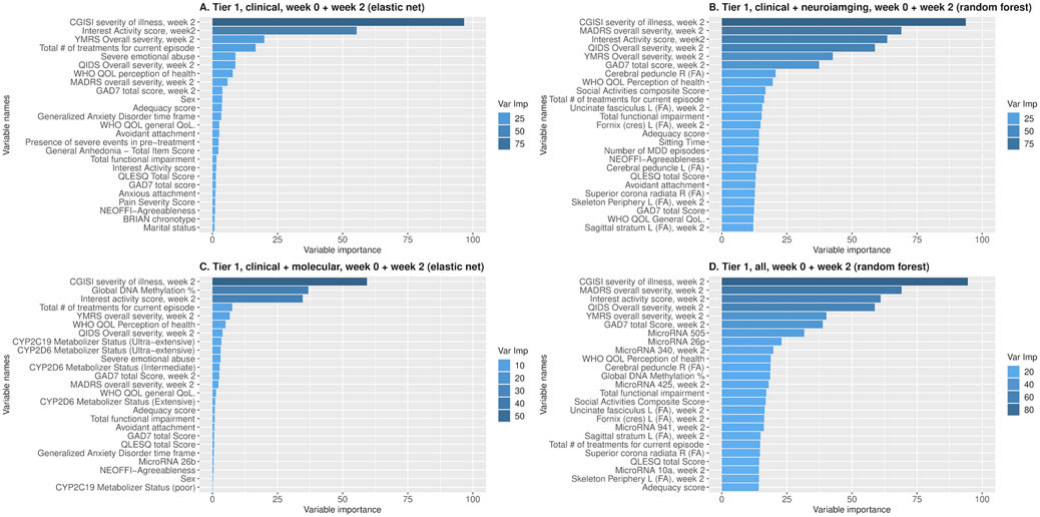
Variable importance of the most predictive models with the highest mean balanced accuracy among all of the 210 models including (A) elastic net model using tier 1 clinical variables in week 0 + week 2; (B) random forest model using tier 1 clinical + neuroimaging variables in week 0 + week 2; (C) elastic net model using tier 1 clinical + molecular variables in week 0 + week 2; (D) random forest model using all tier 1 variables in week 0 + week 2.

When designing the development of the prediction model, we followed the current recommendations to reduce the risk of bias (ROB) and overfitting (Moons et al., 2019; Ranstam, Cook, & Collins, 2016; Wolff et al., 2019). To ensure complete separation of training and testing sets and minimize the ROB or over-optimism, we applied a fully nested cross-validation framework, with all procedures, including the imputation of missing values, performed separately in training and testing sets within each fold of the outer cross-validation loop (Fig. 1). For each combination of predictor modality (clinical, molecular, neuroimaging, clinical + molecular, clinical + neuroimaging, molecular + neuroimaging, and all three modalities), predictor tier (tier 1, and tier 2), predictor measurement time (baseline only, the combination of baseline and week 2), and machine learning method, we completed 100 repetitions of nested cross-validation (inner fivefold cross-validation and outer threefold cross-validation). We applied five machine learning methods with potentially complementary advantages: a penalized multiple regression (hyperparameter tuned elastic net) (Kuhn, 2020), two tree-based methods [random forests and gradient boosting (GBM)], support vector machines (SVM) with radial basis kernel (Kuhn, 2020; Meyer et al., 2021), and Bayesian network analysis (Naïve Bayes) (Meyer et al., 2021). We applied each machine learning method with and without feature selection using CAT scores (correlation-adjusted *t*-scores) in the sda package (Ahdesmäki, Zuber, Gibb, & Strimmer, 2015) to select the top 25 features in each training set. To prevent information leakage, feature selection was carried out in the training set only within each fold of each repeat of the outer cross-validation. Elastic net, GBM, and random forests have additional embedded feature selection, which were also nested and restricted to the training set within each fold and repeat. In total, we developed and evaluated 210 models representing combinations of the modality, predictor tier, predictor measurement time, machine learning method, and CAT feature selection. In each case, we predicted a categorically defined response. Since the outcome distribution was balanced and there was no *a priori* assumption of differential penalty for false-positive and false-negative predictions, we quantified prediction as balanced accuracy in testing sets the nested cross-validation. We report the mean balanced accuracy and the range of balanced accuracy across the 100 repeats of nested cross-validation. We examine variable importance to evaluate the contribution of specific predictors in models with the highest predictive accuracy (Fig. 2; online Supplementary Figs S3–S6). The Prediction model Risk Of Bias Assessment Tool (PROBAST) (Moons et al., 2019; Wolff et al., 2019) is reported in online Supplementary Table S4. Since the model was developed without external validation, the overall ROB was considered high even though other domains showed low ROB.

## Results

### Prediction of response from tier 1 baseline predictors

**Fig. 3.**
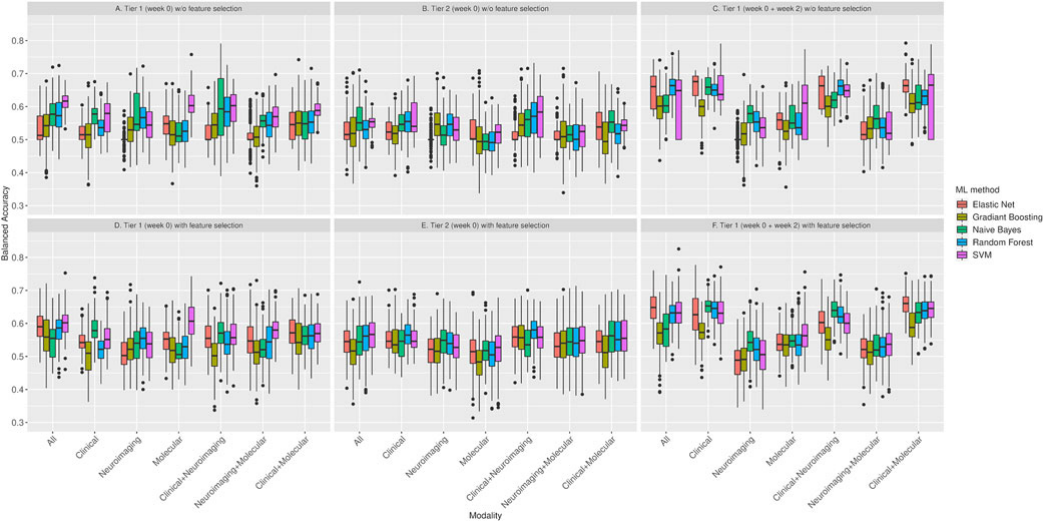
Balanced accuracy of 210 machine learning models for tier 1 data (week 0) without feature selection (*A*), and with feature selection (*D*); tier 2 data (week 0) without feature selection (*B*), and with feature selection (*E*); tier 1 data (week 0 + week 1) without feature selection (*C*), and with feature selection (*F*). Note that in each box plot, the lower and upper whiskers indicate the smallest value within 1.5 times the interquartile range below the 25th percentile to the largest value within 1.5 times the interquartile range above the 75th percentile, the lower and upper hinges indicate the 25th percentile and 75th percentile respectively. The middle line inside the box is 50th percentile (median), and the dots are outside values that are >1.5 times and <3 times the interquartile range beyond either of box.

**Fig. 4.**
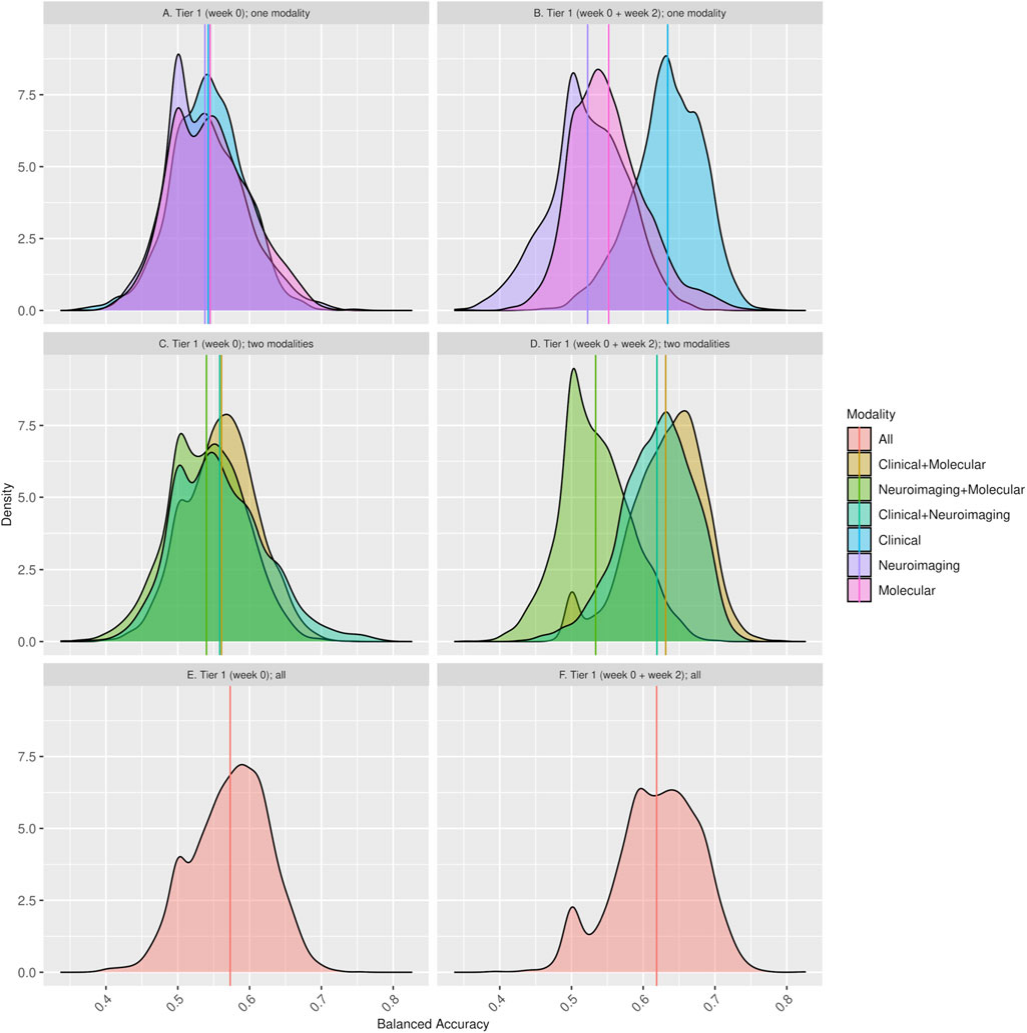
Distribution of balanced accuracy estimates across (*A*) tier 1 (week 0) for one modality at a time; (*C*) combinations of two modalities; (*E*) the combination of three modalities, and (*B*) tier 1 (week 0 + week 2) for one modality at a time; (*D*) combinations of two modalities; (*F*) the combination of three modalities. The solid vertical lines represent the mean balanced accuracy of each distribution.

Up to 134 predictors across the three modalities were included in tier 1 analyses. Across 70 machine learning models using seven combinations of predictor modality and five machine learning methods, each with and without feature selection, we predicted response with a mean balanced accuracy of 0.55 (median 0.55, Fig. 3; Table 1; online Supplementary Table S5). There was a gradient of increasing prediction accuracy with the inclusion of multiple predictor modalities. Using a single predictor modality led to a mean accuracy of 0.542 (95% CI 0.541–0.543; s.e. = 0.000562), the combination of two predictor modalities predicted a mean accuracy of 0.553 (95% CI 0.552–0.554; s.e. = 0.000597), the combination of features across all three modalities predicted response with a mean balanced accuracy of 0.573 across methods (95% CI 0.571–0.575; s.e. = 0.000971). The proportion of individuals misclassified in terms of response to antidepressant treatment decreased from 46.9% with change prediction to 44.8% with the best performing set of models using baseline clinical predictors, and 37.4% with the best set of models including all three data modalities. The distributions of balanced accuracy estimates for one, two, and three baseline data modalities are illustrated in Fig 4*A*, *C*, *E*.

Of the machine learning methods, SVM reported the highest mean accuracy (0.58). Feature selection did not affect reported accuracy (mean 0.55 with and 0.55 without feature selection). The highest balanced accuracy of 0.62 was seen with SVM without feature selection using a combination of predictors from all three modalities. Molecular (global DNA methylation, plasma cholesterol, microRNA 26p), clinical (functional impairment, anhedonia), and neuroimaging (fractional anisotropy in several white matter regions) variables all contributed to the prediction (online Supplementary Fig. S3). The most accurate predictive models for each predictor combination are described in Table 1. Receiver operating characteristic (ROC) curves for the most predictive [highest area under the curve (AUC)] models are depicted in online Supplementary Fig. S7*A*–*G*.

### Prediction of response from tier 2 baseline predictors

Tier 2 analyses involved a more than eightfold increase in the number of predictors across modalities compared to tier 1 (1152 *v.* 134 features, respectively). The 70 machine learning models covering all combinations of predictor modality and methods predicted response with a mean balanced accuracy of 0.54 (median 0.54, interquartile range 0.52–0.55; Fig. 3; Table 1; online Supplementary Table S5). Using a single predictor modality led to a mean accuracy of 0.528 (95% CI 0.527–0.529; s.e. = 0.000538), the combination of two predictor modalities achieved a mean accuracy of 0.541 (95% CI 0.540–0.542; s.e. = 0.000581), and a combination of features across all three modalities predicted response with a mean balanced accuracy of 0.543 (95% CI 0.541–0.545; s.e. = 0.000942).

The choice of the machine learning method was unrelated to prediction accuracy (Fig. 3; online Supplementary Table S5). Use of CAT score feature selection was associated with a modest increase in accuracy among tier 2 analyses (mean balanced accuracy 0.54 with and 0.53 without feature selection). The highest balanced accuracy of 0.58 was seen using random forest with CAT score feature selection and the combination of clinical and neuroimaging data. When all three modalities were included, the most important contributors to response prediction were polygenic scores for insomnia and educational achievement, suicidal ideation, reduced appetite, plasma cholesterol, and methylation at several loci (online Supplementary Fig. S3). Across modalities and methods, tier 2 analyses consistently achieved slightly lower prediction accuracy than tier 1 analyses (Fig. 3; online Supplementary Table S5). ROC curves for the most predictive (highest AUC) models are depicted in online Supplementary Fig. S8*A*–*G*.

### Prediction of response from baseline and week 2 predictors

In the next step, we examined how additional measurements early in the course of treatment (at week 2) improved prediction. Since the inclusion of tier 2 variables reduced prediction accuracy, we focused this stage of analysis on tier 1 predictors. We tested 70 machine learning models with up to 80 variables measured at week 2 added to baseline tier 1 predictors.

These models predicted response with a mean balanced accuracy of 0.59 (median 0.60, interquartile range 0.54–0.63; Fig. 3; Table 1; online Supplementary Table S5). There was a gradient of increasing prediction accuracy with the inclusion of multiple predictor modalities. Using a single predictor modality led to a mean accuracy of 0.570 (95% CI 0.568–0.571; s.e. = 0.000738), the combination of two predictor modalities predicted a mean accuracy of 0.595 (95% CI 0.593–0.596; s.e. = 0.000703), the inclusion of baseline and week 2 features across all three modalities predicted response with a mean balanced accuracy of 0.619 (95% CI 0.617–0.621; s.e. = 0.001070) across methods. The distributions of balanced accuracy estimates for combinations of baseline and week 2 measurements are illustrated in Fig. 4*B*, *D*, *F*.

The decisive factor was the inclusion of clinical modality variables measured at week 2. The most accurate predictive models included an elastic net model using clinical predictors without feature selection, and a random forest model using data from all three modalities without additional feature selection, both achieving a balanced accuracy of 0.66 (Fig. 3; Table 1). In the most accurate models, clinical variables (clinical global impression, interest-activity symptoms score, and total depression severity scores from clinician-rated and self-report instruments) measured at week 2 contributed most to the prediction of response (online Supplementary Fig. S6). ROC curves for the most predictive (highest AUC) models are depicted in online Supplementary Fig. S9*A*–*G*.

## Discussion

In a medium-sized richly assessed sample of patients with MDD, we show that multimodal assessment improves the prediction of antidepressant treatment outcomes. The improvement in prediction accuracy with the inclusion of molecular and neuroimaging information is modest. The addition of week 2 measurements leads to a more substantial improvement in prediction accuracy.

The strength of prediction should be interpreted in the context of existing literature and the known relationship between study quality and prediction accuracy. A recent meta-analysis found that studies with adequate methodology (sample size over 100 and clear separation of training and testing sets) report prediction with lower accuracy than studies with small samples or inadequate separation of training and testing sets (Sajjadian et al., 2021). In addition, response is the hardest outcome to predict, because it is uncorrelated with baseline severity (Coley et al., 2020). Studies with adequate methodology reported mean prediction accuracies of 0.69, 0.60, and 0.56 for the prediction of treatment resistance, remission, and response respectively (Sajjadian et al., 2021). The mean accuracy of prediction across the range of models in the present study (0.56) is comparable to reports in prior studies that used adequate quality methods (Sajjadian et al., 2021). The use of baseline clinical measures helped predict one additional response per 50 patients compared to chance prediction. This level of accuracy is above chance but does not meet the standards required for meaningful clinical application (Dinga et al., 2018; Uher, Tansey, Malki, & Perlis, 2012b). The use of baseline clinical, molecular, and neuroimaging measures helped predict one additional response per 10 patients compared to chance prediction. This still falls short of clinical significance, especially if blood and neuroimaging biomarkers are not easily accessible, but it is a step toward clinically meaningful multimodal prediction as measurements become more accessible and algorithms improve. In a prior study, the addition of molecular genetic variables to clinical features increased prediction accuracy compared to using clinical features alone (Iniesta et al., 2018, 2016), while others reported highly accurate predictions of depression treatment outcomes from neuroimaging variables (Cash et al., 2019; Williams et al., 2015). However, these studies had small samples and did not answer the question as to whether the higher accuracy is due to the unique predictive value of neuroimaging or a result of overfitting (Sajjadian et al., 2021). The present study is the first one to combine clinical, molecular, and neuroimaging features in a single sample. In the present study, we found a gradient of increasing accuracy with the inclusion of additional modalities of measurement. Both mean accuracy and best predictive model accuracy were highest when clinical, molecular, and neuroimaging predictors were combined. This finding supports prior findings on the added benefits of molecular (Iniesta et al., 2018) and neuroimaging (Williams et al., 2015) and extends them to combinations of clinical, molecular, and neuroimaging data. The lack of prediction increase in accuracy with tier 2 variables suggests that the improvement is due to multiple data modalities rather than just a greater number of variables. However, the degree of prediction accuracy improvement in our data was not as large as that previously reported for molecular (Iniesta et al., 2018) or neuroimaging (Cash et al., 2019; Williams et al., 2015). A key decision in designing a predictive model is the number of predictors (features) to be included, relative to the number of participants (observations) that are available. While common recommendations in predictive modeling suggest limiting the number of predictors so that a minimum number of observations per predictor is available, a recent meta-analysis found no relationship between the feature-to-observations ratio and reported prediction accuracy (Sajjadian et al., 2021). The present study contributes to informing the optimal number of predictors in two ways. First, we observed no improvement in predictive accuracy with tier 2 compared to tier 1. Tier 1 models with 31-to-134 predictors achieved more accurate predictions than tier 2 models with 194–1152 predictors. Second, the addition of predictor modalities or time points within tier 1 did improve prediction accuracy. Third, feature selection was associated with improved accuracy in tier 2, but not in tier 1. Together, the findings suggest that when predicting treatment outcomes, a predictive model can make use of a number of features smaller than or similar to the number of participants. When the number of predictors exceeds the number of participants, the predictive model development becomes less efficient. The strength of correlations among predictors and the degree of association between each predictor and the outcome in different datasets may modify these conclusions.

Response to antidepressants evolves over 6–8 weeks, but early changes in symptoms within the first 2 weeks of treatment are predictive of longer-term outcomes (Szegedi et al., 2009). In the present study, the addition of measures obtained 2 weeks after treatment initiation led to a striking improvement in prediction accuracy. This improvement in accuracy exceeded the benefits of multimodal measurement at baseline. While multimodal models retained a degree of advantage after the inclusion of week 2, clinical measures obtained at 2 weeks made the most substantial contribution to the improved prediction. This marked improvement in prediction is consistent with other reports. Machine learning studies that included week 2 data (Chekroud et al., 2016; Nie et al., 2018) reported more accurate predictions than those that used baseline predictors only (Chekroud et al., 2016). A recent study reported high accuracy when data obtained 4 weeks after the onset of treatment were included as predictors (Athreya et al., 2021). These results seem to point to a greater value of initial treatment data compared to extensive baseline assessments. However, the clinical value of prediction and its potential to change treatment course is diminishing with time from baseline as the trajectory of response is already becoming apparent and many clinicians decide to adjust treatment accordingly (Browning et al., 2021).

The present study benefits from rich multimodal assessment and standardized protocols. However, the results should be interpreted regarding limitations related to the sample size. The present sample, although larger than previously reported multimodal studies, is not large enough to optimally support the learning or validation of complex prediction models. Given the size of the available data set, we opted for nested cross-validation that uses all parts of the dataset as both training and testing sets in different validation loops while retaining a strict separation of the training and testing sets. The optimal validation strategy includes an additional step of external validation (Chekroud et al., 2021). Ideally, the external validation should occur in a dataset that was not available at the time of model development. CAN-BIND is presently collecting a new dataset with treatment and assessment protocols closely matching those used in the present study. This will present the first opportunity to replicate results in a sample that is strictly external to the model development and yet is fully comparable.

In conclusion, a range of machine learning analyses suggests that a combination of clinical data with neuroimaging and molecular biomarkers improves the prediction of antidepressant treatment outcomes over single modality measurement. Larger samples will be required to scale the present work up and use the full potential of rich multimodal measurements to achieve clinically meaningful response prediction.

## Supplementary material

For supplementary material accompanying this paper visit https://doi.org/10.1017/S0033291722002124. click here to view supplementary material
