# Supplementary material for: Prediction of depression treatment outcome from multimodal data: a CAN-BIND-1 report
Source: Psychol Med. 2022 Aug 25;53(12):5374–84. doi: 10.1017/S0033291722002124 (PMC10482706; doi:10.1017/S0033291722002124)

**SUPPLEMENTARY APPENDIX**

Table of Contents:

Results 2

Table S1 4

Table S2 5

Table S3 6

Table S4 13

Table S5 14

Figure S1 19

Figure S2 20

Figure S3 21

Figure S4 22

Figure S5 23

Figure S6 24

Figure S7 25

Figure S8 26

Figure S9 27

# RESULTS

**Prediction of response using Tier 1 dataset (week 0)**

Across each set of 3000 extracted results (100 repetition of 3-fold cross-validation × 5 types of models × 2 (with and without feature selection)); the balanced accuracy ranged from 0.36 to 0.74 (mean=0.54, median = 0.54) for clinical data; 0.37 to 0.76 (mean=0.55, median = 0.54) for molecular data; 0.40 to 0.72 (mean=0.54, median = 0.53) for neuroimaging data; 0.34 to 0.79 (mean=0.56, median = 0.55) for clinical + neuroimaging data; 0.36 to 0.73 (mean=0.54, median = 0.54) for neuroimaging + molecular data; 0.40 to 0.74 (mean=0.56, median = 0.56) for clinical + molecular data; and 0.39 to 0.75 (mean=0.57, median 0.58) for clinical + neuroimaging + molecular data.

**Prediction of response using Tier 2 dataset (week 0)**

Across each set of 3000 extracted results (100 repetition of 3-fold cross-validation × 5 types of models × 2 (with and without feature selection))extracted results; balanced accuracy ranged from 0.39 to 0.70 (mean=0.55 , median = 0.54) for clinical data; 0.31 to 0.71 (mean=0.51, median = 0.51) for molecular data; 0.38 to 0.70 (mean=0.53, median = 0.53) for neuroimaging data; 0.40 to 0.73 (mean=0.55, median =0.55) for clinical + neuroimaging data; 0.34 to 0.72 (mean=0.53, median =0.53 ) for neuroimaging + molecular data; 0.37 to 0.71 (mean=0.54, median = 0.54) for clinical + molecular data; and 0.36 to 0.73 (mean= 0.54, median = 0.54) for clinical + neuroimaging + molecular data .

**Prediction of response using Tier 1 dataset (week 0 + week 2)**

Across each set of 3000 extracted results (100 repetition of 3-fold cross-validation × 5 types of models × 2 (with and without feature selection)), balanced accuracy ranged from 0.44 to 0.79 (mean=0.63 , median =0.64 ) for clinical data; 0.36 to 0.77 (mean=0.55, median =0.55) for molecular data; 0.34 to 0.70 (mean=0.52, median =0.52 ) for neuroimaging data; 0.43 to 0.75 (mean=0.62, median =0.62) for clinical + neuroimaging data; 0.35 to 0.70 (mean=0.53 , median =0.53) for neuroimaging + molecular data; 0.47 to 0.79 (mean=0.63, median =0.64 ) for clinical + molecular data; and 0.39 to 0.83 (mean=0.62 , median = 0.62) for clinical + neuroimaging + molecular data.

**TABLES AND FIGURES**

**Table S1:** Comparison of participants who did and did not contribute to analyses.

|  |  |  | **No outcomes** | |  | **Included in analyses** | |  | **Test of difference** | |
| --- | --- | --- | --- | --- | --- | --- | --- | --- | --- | --- |
|  |  |  | n=19 |  |  | n = 192 |  |  |  |  |
|  |  |  |  |  |  |  |  |  |  |  |
|  |  |  | Count | % |  | Count | % |  | chi^2^ | p |
|  | Sex: Female |  | 15 | 78.95% |  | 118 | 61.46% |  | 2.27 | 0.132 |
|  | Married / cohabiting |  | 4 | 21.05% |  | 51 | 26.56% |  | 0.27 | 0.602 |
|  | Employed / student |  | 11 | 57.89% |  | 120 | 62.50% |  | 0.16 | 0.693 |
|  | Ethnicity: White |  | 12 | 63.16% |  | 138 | 71.88% |  | 0.64 | 0.424 |
|  | Comorbidity: Anxiety disorder |  | 10 | 52.63% |  | 89 | 46.35% |  | 0.27 | 0.601 |
|  | Comorbidity: Substance use |  | 0 | 0.00% |  | 8 | 4.17% |  | 0.82 | 0.364 |
|  |  |  |  |  |  |  |  |  |  |  |
|  |  |  |  |  |  |  |  |  |  |  |
|  |  |  | Mean | S.D. |  | Mean | S.D. |  | t | p |
|  | Age (years) |  | 34.5 | 11.7 |  | 35.4 | 12.8 |  | -0.30 | 0.765 |
|  | Baseline MADRS (total score) |  | 28.3 | 6.4 |  | 30.0 | 5.5 |  | -1.27 | 0.204 |
|  | Baseline QIDS-SR (total score) |  | 14.3 | 4.1 |  | 15.9 | 4.1 |  | -1.61 | 0.110 |

**Table S2:** Number of variables in tier 1 and tier 2 dataset

| **Analysis** | **Modality** | **Predictive week** | **Number of variables** |
| --- | --- | --- | --- |
| Tier 1 | All | Week 0 | 134 |
| Tier 1 | Clinical | Week 0 | 47 |
| Tier 1 | Molecular | Week 0 | 31 |
| Tier 1 | Neuroimaging | Week 0 | 56 |
| Tier 1 | All | Week 2 | 80 |
| Tier 1 | Clinical | Week 2 | 8 |
| Tier 1 | Molecular | Week 2 | 21 |
| Tier 1 | Neuroimaging | Week 2 | 51 |
| Tier 2 | All | Week 0 | 1152 |
| Tier 2 | Clinical | Week 0 | 194 |
| Tier 2 | Molecular | Week 0 | 733 |
| Tier 2 | Neuroimaging | Week 0 | 225 |

**Table S3:** Full description of tier 1 variables

| **Category** | **Modality of Data** | **Analysis** | **Scale** | **Variable** | **Type of variable** | **Variable name in data excel file** |
| --- | --- | --- | --- | --- | --- | --- |
| Clinical | Demographics | Tier 1 | Demographics | Age | Continuous | AGE |
| Clinical | Demographics | Tier 1 | Demographics | Sex | Categorical | SEX |
| Clinical | Demographics | Tier 1 | Demographics | Marital Status | Categorical | MRTL_STATUS |
| Clinical | Demographics | Tier 1 | Body Mass Index | BMI | Continuous | BL_BMI |
| Clinical | Clinical History | Tier 1 | Psychiatric History | Number of MDD episodes | Continuous | PSYHIS_MDE_NUM_VERIFIED |
| Clinical | Clinical History | Tier 1 | Psychiatric History | Episode duration | Continuous | PSYHIS_MDE_EP_DUR_MO |
| Clinical | Clinical History | Tier 1 | Antidepressant Treatment History Form | Adequacy score | Continuous | ATHF_CUR_ADEQUATE |
| Clinical | Clinical History | Tier 1 | Antidepressant Treatment History Form | Total # of treatments for current episode | Continuous | ATHF_CUR_TOTAL |
| Clinical | Clinical History | Tier 1 | The Mini-International Neuropsychiatric Interview | Generalized Anxiety Disorder time frame | Continuous | MINI_GAD_TIME |
| Clinical | Clinician Rated Study Outcomes | Tier 1 | Montgomery–Åsberg Depression Rating Scale | MADRS overall severity | Categorical | MADRS_TOT_PRO_RATED |
| Clinical | Clinician Rated Study Outcomes | Tier 1 | Clinical Global Impression | Severity of illness | Continuous | CGISI_SEV_OF_ILL |
| Clinical | Clinician Rated Study Outcomes | Tier 1 | Young Mania Rating Scale | Overall severity | Continuous | YMRS_OVERL_SEVTY |
| Clinical | Clinician Rated Study Outcomes | Tier 1 | Depression Inventory Development | General Anhedonia - Total Item Score | Continuous | DID_ANHDN_TOT |
| Clinical | Clinician Rated Study Outcomes | Tier 1 | Depression Inventory Development | Social Activities - Composite Score | Continuous | DID_SOCIAL_COMP_TOT |
| Clinical | Clinician Rated Study Outcomes | Tier 1 | Depression Inventory Development | Information Processing-Cognitive Slowing - Composite Score | Continuous | DID_COGNTV_COMP_TOT |
| Clinical | Clinical History | Tier 1 | Childhood Experience of Care and Abuse | Severe emotional abuse (any antipathy or neglect) | Categorical | MCECA_SVEM |
| Clinical | Self-Reports Clinical Outcomes | Tier 1 | CNS Vital Signs | Neuro-Cognitive Index - Average (mean) of five domain scores: composite memory, psychomotor speed, reaction time, complex attention, and cognitive flexibility; representing the global score of neurocognition | Continuous | vs7_nci_ss |
| Clinical | Demographics | Tier 1 | CNS Vital Signs | Heaton's Corrected Years of Education | Continuous | corrEDUC |
| Clinical | Self-Reports Clinical Outcomes | Tier 1 | Behavioral Avoidance/Inhibition Scales | Reward Responsiveness (RR) | Continuous | BISBAS_RR_Tot |
| Clinical | Self-Reports Clinical Outcomes | Tier 1 | Behavioral Avoidance/Inhibition Scales | Drive (DR) | Continuous | BISBAS_DR_Tot |
| Clinical | Self-Reports Clinical Outcomes | Tier 1 | Brief Pain Inventory | Pain Severity Score | Continuous | BPI_CMPS |
| Clinical | Self-Reports Clinical Outcomes | Tier 1 | Brief Pain Inventory | Pain Interference Score | Continuous | BPI_PICS |
| Clinical | Self-Reports Clinical Outcomes | Tier 1 | The Biological Rhythms Interview for Assessment in Neuropsychiatry | Total Score | Continuous | BRIAN_Tot |
| Clinical | Self-Reports Clinical Outcomes | Tier 1 | The Biological Rhythms Interview for Assessment in Neuropsychiatry | Chronotype | Continuous | BRIAN_Chrono_Tot |
| Clinical | Self-Reports Clinical Outcomes | Tier 1 | Dimensional Anhedonia Rating Scale | Total Score | Continuous | DARS_Tot |
| Clinical | Self-Reports Clinical Outcomes | Tier 1 | The Experiences in Close Relationships-Revised Questionnaire | Anxious attachment | Continuous | ECRR_Attachment.related_anxiety |
| Clinical | Self-Reports Clinical Outcomes | Tier 1 | The Experiences in Close Relationships-Revised Questionnaire | Avoidant attachment | Continuous | ECRR_Attachment.related_avoidance |
| Clinical | Self-Reports Clinical Outcomes | Tier 1 | Generalized Anxiety Disorder 7-item scale | GAD7 Total Score | Continuous | GAD7_Tot |
| Clinical | Self-Reports Clinical Outcomes | Tier 1 | Hypomania/Mania Symptom Checklist 32 | HCL32 Total Score | Continuous | HCL_Total |
| Clinical | Self-Reports Clinical Outcomes | Tier 1 | The International Physical Activity Questionnaire | Vigorous Physical Activity | Continuous | vigorous |
| Clinical | Self-Reports Clinical Outcomes | Tier 1 | The International Physical Activity Questionnaire | Sitting Time | Continuous | sitting |
| Clinical | Self-Reports Clinical Outcomes | Tier 1 | The International Physical Activity Questionnaire | Overall Activity | Continuous | activity |
| Clinical | Self-Reports Clinical Outcomes | Tier 1 | Interest Activity Symptom Dimension from MADRS and QIDS | Interest Activity Symptom Dimension standardized score | Continuous | zblintact |
| Clinical | Self-Reports Clinical Outcomes | Tier 1 | The Life Events and Difficulties Schedule | Presence of severe events in pre-treatment | Categorical | LEDS_SEV16P |
| Clinical | Self-Reports Clinical Outcomes | Tier 1 | The NEO Five-Factor Inventory | NEOFFI-Neuroticism | Continuous | NEOFFI_Neuroticism |
| Clinical | Self-Reports Clinical Outcomes | Tier 1 | The NEO Five-Factor Inventory | NEOFFI-Extraversion | Continuous | NEOFFI_Extraversion |
| Clinical | Self-Reports Clinical Outcomes | Tier 1 | The NEO Five-Factor Inventory | NEOFFI-Agreeableness | Continuous | NEOFFI_Agreeableness |
| Clinical | Self-Reports Clinical Outcomes | Tier 1 | The Pittsburgh Sleep Quality Index | Global PSQI Score | Continuous | PSQI_TOT |
| Clinical | Self-Reports Clinical Outcomes | Tier 1 | Quick Inventory of Depressive Symptomatology - Self Report | QIDS Overall severity | Continuous | QIDS_OVERL_SEVTY |
| Clinical | Self-Reports Clinical Outcomes | Tier 1 | Quality of Life Enjoyment and Satisfaction Questionnaire – Short Form | QLESQ Total Score | Continuous | QLESQ_Tot |
| Clinical | Self-Reports Clinical Outcomes | Tier 1 | Sheehan Disability Scale | Total functional impairment | Continuous | SDS_Tot |
| Clinical | Self-Reports Clinical Outcomes | Tier 1 | Sexual Side Effect Scale | SexFX - Function score | Continuous | SEXFX_FUNCTION |
| Clinical | Self-Reports Clinical Outcomes | Tier 1 | Sexual Side Effect Scale | SexFX - Global Sexual Impression | Continuous | SEXFX_GSI |
| Clinical | Self-Reports Clinical Outcomes | Tier 1 | Snaith–Hamilton Pleasure Scale | Anhedonia | Continuous | SHAPS_Tot |
| Clinical | Self-Reports Clinical Outcomes | Tier 1 | Seasonality Pattern Assessment Questionnaire | Global seasonality score | Continuous | SPAQ_Global_Seasonality |
| Clinical | Self-Reports Clinical Outcomes | Tier 1 | World Health Organization Quality of Life Instruments | WHO QOL General QoL. | Continuous | WHO_QOL_General_QoL. |
| Clinical | Self-Reports Clinical Outcomes | Tier 1 | World Health Organization Quality of Life Instruments | WHO QOL Perception of health. | Continuous | WHO_QOL_Perception_of_health. |
| Neuroimaging | Structural Magnetic Resonance Imaging | Tier 1 | Volumetric data | Estimated Total Intra-Cranial Volume | Continuous | etiv |
| Neuroimaging | Structural Magnetic Resonance Imaging | Tier 1 | Volumetric data | Brain Segmentation Volume Without Ventricles | Continuous | bsvwv |
| Neuroimaging | Structural Magnetic Resonance Imaging | Tier 1 | Volumetric data | Hippocampus - manual - Left Hemisphere | Continuous | bsl_manual_lh |
| Neuroimaging | Structural Magnetic Resonance Imaging | Tier 1 | Volumetric data | Hippocampus - manual - Right Hemisphere | Continuous | bsl_manual_rh |
| Neuroimaging | Structural Magnetic Resonance Imaging | Tier 1 | Volumetric data | Total brain volume | Continuous | Total_Brain_Volume |
| Neuroimaging | Diffusion-Weighted Magnetic Resonance Imaging | Tier 1 | White Matter structure | Genu of corpus callosum - fractional anisotropy (FA) | Continuous | gcc_FA |
| Neuroimaging | Diffusion-Weighted Magnetic Resonance Imaging | Tier 1 | White Matter structure | Body of corpus callosum - fractional anisotropy (FA) | Continuous | bcc_FA |
| Neuroimaging | Diffusion-Weighted Magnetic Resonance Imaging | Tier 1 | White Matter structure | Splenium of corpus callosum - fractional anisotropy (FA) | Continuous | scc_FA |
| Neuroimaging | Diffusion-Weighted Magnetic Resonance Imaging | Tier 1 | White Matter structure | Superior cerebellar peduncle R - fractional anisotropy (FA) | Continuous | scp_r_FA |
| Neuroimaging | Diffusion-Weighted Magnetic Resonance Imaging | Tier 1 | White Matter structure | Superior cerebellar peduncle L - fractional anisotropy (FA) | Continuous | scp_l_FA |
| Neuroimaging | Diffusion-Weighted Magnetic Resonance Imaging | Tier 1 | White Matter structure | Cerebral peduncle R - fractional anisotropy (FA) | Continuous | cp_r_FA |
| Neuroimaging | Diffusion-Weighted Magnetic Resonance Imaging | Tier 1 | White Matter structure | Cerebral peduncle L - fractional anisotropy (FA) | Continuous | cp_l_FA |
| Neuroimaging | Diffusion-Weighted Magnetic Resonance Imaging | Tier 1 | White Matter structure | Anterior limb of internal capsule R - fractional anisotropy (FA) | Continuous | alic_r_FA |
| Neuroimaging | Diffusion-Weighted Magnetic Resonance Imaging | Tier 1 | White Matter structure | Anterior limb of internal capsule L - fractional anisotropy (FA) | Continuous | alic_l_FA |
| Neuroimaging | Diffusion-Weighted Magnetic Resonance Imaging | Tier 1 | White Matter structure | Posterior limb of internal capsule R - fractional anisotropy (FA) | Continuous | plic_r_FA |
| Neuroimaging | Diffusion-Weighted Magnetic Resonance Imaging | Tier 1 | White Matter structure | Posterior limb of internal capsule L - fractional anisotropy (FA) | Continuous | plic_l_FA |
| Neuroimaging | Diffusion-Weighted Magnetic Resonance Imaging | Tier 1 | White Matter structure | Retrolenticular part of internal capsule R - fractional anisotropy (FA) | Continuous | rlic_r_FA |
| Neuroimaging | Diffusion-Weighted Magnetic Resonance Imaging | Tier 1 | White Matter structure | Retrolenticular part of internal capsule L - fractional anisotropy (FA) | Continuous | rlic_l_FA |
| Neuroimaging | Diffusion-Weighted Magnetic Resonance Imaging | Tier 1 | White Matter structure | Anterior corona radiata R - fractional anisotropy (FA) | Continuous | acr_r_FA |
| Neuroimaging | Diffusion-Weighted Magnetic Resonance Imaging | Tier 1 | White Matter structure | Anterior corona radiata L - fractional anisotropy (FA) | Continuous | acr_l_FA |
| Neuroimaging | Diffusion-Weighted Magnetic Resonance Imaging | Tier 1 | White Matter structure | Superior corona radiata R - fractional anisotropy (FA) | Continuous | scr_r_FA |
| Neuroimaging | Diffusion-Weighted Magnetic Resonance Imaging | Tier 1 | White Matter structure | Superior corona radiata L - fractional anisotropy (FA) | Continuous | scr_l_FA |
| Neuroimaging | Diffusion-Weighted Magnetic Resonance Imaging | Tier 1 | White Matter structure | Posterior corona radiata R - fractional anisotropy (FA) | Continuous | pcr_r_FA |
| Neuroimaging | Diffusion-Weighted Magnetic Resonance Imaging | Tier 1 | White Matter structure | Posterior corona radiata L - fractional anisotropy (FA) | Continuous | pcr_l_FA |
| Neuroimaging | Diffusion-Weighted Magnetic Resonance Imaging | Tier 1 | White Matter structure | Posterior thalamic radiation (include optic radiation) R - fractional anisotropy (FA) | Continuous | ptr_r_FA |
| Neuroimaging | Diffusion-Weighted Magnetic Resonance Imaging | Tier 1 | White Matter structure | Posterior thalamic radiation (include optic radiation) L - fractional anisotropy (FA) | Continuous | ptr_l_FA |
| Neuroimaging | Diffusion-Weighted Magnetic Resonance Imaging | Tier 1 | White Matter structure | Sagittal stratum (include inferior longitudinal fasciculus and inferior fronto-occipital fasciculus) R - fractional anisotropy (FA) | Continuous | ss_r_FA |
| Neuroimaging | Diffusion-Weighted Magnetic Resonance Imaging | Tier 1 | White Matter structure | Sagittal stratum (include inferior longitudinal fasciculus and inferior fronto-occipital fasciculus) L - fractional anisotropy (FA) | Continuous | ss_l_FA |
| Neuroimaging | Diffusion-Weighted Magnetic Resonance Imaging | Tier 1 | White Matter structure | External capsule R - fractional anisotropy (FA) | Continuous | ec_r_FA |
| Neuroimaging | Diffusion-Weighted Magnetic Resonance Imaging | Tier 1 | White Matter structure | External capsule L - fractional anisotropy (FA) | Continuous | ec_l_FA |
| Neuroimaging | Diffusion-Weighted Magnetic Resonance Imaging | Tier 1 | White Matter structure | Cingulum (cingulate gyrus) R - fractional anisotropy (FA) | Continuous | cgc_r_FA |
| Neuroimaging | Diffusion-Weighted Magnetic Resonance Imaging | Tier 1 | White Matter structure | Cingulum (cingulate gyrus) L - fractional anisotropy (FA) | Continuous | cgc_l_FA |
| Neuroimaging | Diffusion-Weighted Magnetic Resonance Imaging | Tier 1 | White Matter structure | Cingulum (hippocampus) R - fractional anisotropy (FA) | Continuous | cgh_r_FA |
| Neuroimaging | Diffusion-Weighted Magnetic Resonance Imaging | Tier 1 | White Matter structure | Cingulum (hippocampus) L - fractional anisotropy (FA) | Continuous | cgh_l_FA |
| Neuroimaging | Diffusion-Weighted Magnetic Resonance Imaging | Tier 1 | White Matter structure | Fornix (cres) / Stria terminalis (cannot be resolved with current resolution) R - fractional anisotropy (FA) | Continuous | fx_st_r_FA |
| Neuroimaging | Diffusion-Weighted Magnetic Resonance Imaging | Tier 1 | White Matter structure | Fornix (cres) / Stria terminalis (cannot be resolved with current resolution) L - fractional anisotropy (FA) | Continuous | fx_st_l_FA |
| Neuroimaging | Diffusion-Weighted Magnetic Resonance Imaging | Tier 1 | White Matter structure | Superior longitudinal fasciculus R - fractional anisotropy (FA) | Continuous | slf_r_FA |
| Neuroimaging | Diffusion-Weighted Magnetic Resonance Imaging | Tier 1 | White Matter structure | Superior longitudinal fasciculus L - fractional anisotropy (FA) | Continuous | slf_l_FA |
| Neuroimaging | Diffusion-Weighted Magnetic Resonance Imaging | Tier 1 | White Matter structure | Superior fronto-occipital fasciculus (could be a part of anterior internal capsule) R - fractional anisotropy (FA) | Continuous | sfo_r_FA |
| Neuroimaging | Diffusion-Weighted Magnetic Resonance Imaging | Tier 1 | White Matter structure | Superior fronto-occipital fasciculus (could be a part of anterior internal capsule) L - fractional anisotropy (FA) | Continuous | sfo_l_FA |
| Neuroimaging | Diffusion-Weighted Magnetic Resonance Imaging | Tier 1 | White Matter structure | Uncinate fasciculus R - fractional anisotropy (FA) | Continuous | unc_r_FA |
| Neuroimaging | Diffusion-Weighted Magnetic Resonance Imaging | Tier 1 | White Matter structure | Uncinate fasciculus L - fractional anisotropy (FA) | Continuous | unc_l_FA |
| Neuroimaging | Diffusion-Weighted Magnetic Resonance Imaging | Tier 1 | White Matter structure | Tapetum R - fractional anisotropy (FA) | Continuous | tap_r_FA |
| Neuroimaging | Diffusion-Weighted Magnetic Resonance Imaging | Tier 1 | White Matter structure | Tapetum L - fractional anisotropy (FA) | Continuous | tap_l_FA |
| Neuroimaging | Diffusion-Weighted Magnetic Resonance Imaging | Tier 1 | White Matter structure | Skeleton Periphery R - fractional anisotropy (FA) | Continuous | sp_r_FA |
| Neuroimaging | Diffusion-Weighted Magnetic Resonance Imaging | Tier 1 | White Matter structure | Skeleton Periphery L - fractional anisotropy (FA) | Continuous | sp_l_FA |
| Neuroimaging | Diffusion-Weighted Magnetic Resonance Imaging | Tier 1 | Volumetric data | Baseline thickness of right medial orbitofrontal | Continuous | w0_rh_medialorbitofrontal |
| Neuroimaging | Diffusion-Weighted Magnetic Resonance Imaging | Tier 1 | Volumetric data | Baseline thickness of left medial orbitofrontal | Continuous | w0_lh_medialorbitofrontal |
| Neuroimaging | Diffusion-Weighted Magnetic Resonance Imaging | Tier 1 | Volumetric data | Baseline thickness of right supramarginal | Continuous | w0_rh_supramarginal |
| Neuroimaging | Diffusion-Weighted Magnetic Resonance Imaging | Tier 1 | Volumetric data | Baseline thickness of left supramarginal | Continuous | w0_lh_supramarginal |
| Neuroimaging | Diffusion-Weighted Magnetic Resonance Imaging | Tier 1 | Volumetric data | Baseline thickness of left pars opercularis | Continuous | w0_lh_parsopercularis |
| Neuroimaging | Diffusion-Weighted Magnetic Resonance Imaging | Tier 1 | Volumetric data | Baseline thickness of right pars opercularis | Continuous | w0_rh_parsopercularis |
| Neuroimaging | Diffusion-Weighted Magnetic Resonance Imaging | Tier 1 | Volumetric data | Baseline thickness of left pericalcarin | Continuous | w0_lh_pericalcarine |
| Neuroimaging | Diffusion-Weighted Magnetic Resonance Imaging | Tier 1 | Volumetric data | Baseline thickness of right pericalcarin | Continuous | w0_rh_pericalcarine |
| Neuroimaging | Diffusion-Weighted Magnetic Resonance Imaging | Tier 1 | Volumetric data | Baseline thickness of right lingual | Continuous | w0_rh_lingual |
| Neuroimaging | Diffusion-Weighted Magnetic Resonance Imaging | Tier 1 | Volumetric data | Baseline thickness of left lingual | Continuous | w0_lh_lingual |
| Molecular | Blood - Analytes | Tier 1 | Inflammation Marker | C-reactive protein levels | Continuous | CRP |
| Molecular | Blood - Genetics | Tier 1 | Percentage of methylated CpG across the genome | Global DNA Methylation % | Continuous | Total_Methylation |
| Molecular | Blood - Pharmacogenetics | Tier 1 | Cytochrome P450 metabolizing enzyme genetic function group | CYP2C19 Metabolizer Status - Extensive metabolizers | Categorical | CYP2C19_Metabolizer_ESCEM |
| Molecular | Blood - Pharmacogenetics | Tier 1 | Cytochrome P450 metabolizing enzyme genetic function group | CYP2C19 Metabolizer Status - Intermediate metabolizers | Categorical | CYP2C19_Metabolizer_ESCIM |
| Molecular | Blood - Pharmacogenetics | Tier 1 | Cytochrome P450 metabolizing enzyme genetic function group | CYP2C19 Metabolizer Status - Poor metabolizers | Categorical | CYP2C19_Metabolizer_ESCPM |
| Molecular | Blood - Pharmacogenetics | Tier 1 | Cytochrome P450 metabolizing enzyme genetic function group | CYP2C19 Metabolizer Status - Ultra-extensive metabolizers | Categorical | CYP2C19_Metabolizer_ESCUM |
| Molecular | Blood - Pharmacogenetics | Tier 1 | Cytochrome P450 metabolizing enzyme genetic function group | CYP2D6 Metabolizer Status - Extensive metabolizers | Categorical | CYP2D6_MetabolizerEM |
| Molecular | Blood - Pharmacogenetics | Tier 1 | Cytochrome P450 metabolizing enzyme genetic function group | CYP2D6 Metabolizer Status - Intermediate metabolizers | Categorical | CYP2D6_MetabolizerIM |
| Molecular | Blood - Pharmacogenetics | Tier 1 | Cytochrome P450 metabolizing enzyme genetic function group | CYP2D6 Metabolizer Status - Poor metabolizers | Categorical | CYP2D6_MetabolizerPM |
| Molecular | Blood - Pharmacogenetics | Tier 1 | Cytochrome P450 metabolizing enzyme genetic function group | CYP2D6 Metabolizer Status - Ultra-extensive metabolizers | Categorical | CYP2D6_MetabolizerUM |
| Molecular | Blood - Genetics | Tier 1 | Micro RNA | Expression of hsa.miR.10a.5p | Continuous | hsa.miR.10a.5p |
| Molecular | Blood - Genetics | Tier 1 | Micro RNA | Expression of hsa.miR.146a.5p | Continuous | hsa.miR.146a.5p |
| Molecular | Blood - Genetics | Tier 1 | Micro RNA | Expression of hsa.miR.146b.5p | Continuous | hsa.miR.146b.5p |
| Molecular | Blood - Genetics | Tier 1 | Micro RNA | Expression of hsa.miR.181a.5p | Continuous | hsa.miR.181a.5p |
| Molecular | Blood - Genetics | Tier 1 | Micro RNA | Expression of hsa.miR.24.3p | Continuous | hsa.miR.24.3p |
| Molecular | Blood - Genetics | Tier 1 | Micro RNA | Expression of hsa.miR.26b.3p | Continuous | hsa.miR.26b.3p |
| Molecular | Blood - Genetics | Tier 1 | Micro RNA | Expression of hsa.miR.340.3p | Continuous | hsa.miR.340.3p |
| Molecular | Blood - Genetics | Tier 1 | Micro RNA | Expression of hsa.miR.425.3p | Continuous | hsa.miR.425.3p |
| Molecular | Blood - Genetics | Tier 1 | Micro RNA | Expression of hsa.miR.505.3p | Continuous | hsa.miR.505.3p |
| Molecular | Blood - Genetics | Tier 1 | Micro RNA | Expression of hsa.miR.6803.3p | Continuous | hsa.miR.6803.3p |
| Molecular | Blood - Genetics | Tier 1 | Micro RNA | Expression of hsa.miR.941 | Continuous | hsa.miR.941 |
| Molecular | Blood - Analytes | Tier 1 | Metabolomics | Triglycerides - Total Plasma | Continuous | bltptg |
| Molecular | Blood - Analytes | Tier 1 | Metabolomics | Cholesterol - Total Plasma | Continuous | bltpch |
| Molecular | Blood - Analytes | Tier 1 | Metabolomics | Cholesterol - LDL | Continuous | blldch |
| Molecular | Blood - Analytes | Tier 1 | Metabolomics | Particle Number - Total Plasma | Continuous | bltbpn |
| Molecular | Blood - Analytes | Tier 1 | Metabolomics | Particle Number LDL | Continuous | blldpn |
| Molecular | Blood - Analytes | Tier 1 | Metabolomics | Triglycerides - VLDL | Continuous | blvltg |
| Molecular | Blood - Analytes | Tier 1 | Metabolomics | Triglycerides - VLDL1 | Continuous | blv1tg |
| Molecular | Urine - Analytes | Tier 1 | Metabolomics | Guanidinoacetic acid | Continuous | blguanidinoaceticacid |
| Molecular | Urine - Analytes | Tier 1 | Metabolomics | Hippuric acid | Continuous | blhippuricacid |
| Molecular | Urine - Analytes | Tier 1 | Metabolomics | Trigonelline | Continuous | bltrigonelline |

**Table S4**: PROBAST checklist for the present study

| **ROB** | | | | **Applicability** | | | | **Overall** | |
| --- | --- | --- | --- | --- | --- | --- | --- | --- | --- |
| **Participants** | **Predictors** | **Outcome** | **Analysis** | **Participants** | **Predictors** | **Outcome** | **Analysis** | **ROB** | **Applicability** |
| + | + | + | + | + | + | + | + | - | + |

PROBAST = Prediction model Risk Of Bias Assessment Tool; ROB = risk of bias.

* + indicates low ROB/low concern regarding applicability; − indicates high ROB/high concern regarding applicability.

**Table S5**: Machine learning results for tier 1 and tier 2 data with response as an outcome

| Analysis | Modality | Predictive week | Machine learning method | Feature selection | # of variables before feature selection | # of variables after feature selection | Sensitivity | Specificity | PPV | NPV | Balanced accuracy |
| --- | --- | --- | --- | --- | --- | --- | --- | --- | --- | --- | --- |
| Tier 1 | All | Week 0 | Naïve Bayes | None | 134 | 134 | 0.59 | 0.56 | 0.56 | 0.61 | 0.58 |
| Tier 1 | All | Week 0 | Elastic net | Embedded | 134 | 134 | 0.30 | 0.77 | 0.55 | 0.56 | 0.54 |
| Tier 1 | All | Week 0 | SVM | None | 134 | 134 | 0.45 | 0.78 | 0.65 | 0.62 | 0.62 |
| Tier 1 | All | Week 0 | GBM | Embedded | 134 | 134 | 0.50 | 0.58 | 0.51 | 0.57 | 0.54 |
| Tier 1 | All | Week 0 | Random forest | Embedded | 134 | 134 | 0.40 | 0.76 | 0.59 | 0.59 | 0.58 |
| Tier 1 | All | Week 0 | Naïve Bayes | CAT score | 134 | 25 | 0.87 | 0.23 | 0.50 | 0.64 | 0.55 |
| Tier 1 | All | Week 0 | Elastic net | Embedded + CAT score | 134 | 25 | 0.57 | 0.62 | 0.57 | 0.62 | 0.59 |
| Tier 1 | All | Week 0 | SVM | CAT score | 134 | 25 | 0.54 | 0.66 | 0.59 | 0.62 | 0.60 |
| Tier 1 | All | Week 0 | GBM | Embedded + CAT score | 134 | 25 | 0.56 | 0.57 | 0.53 | 0.60 | 0.56 |
| Tier 1 | All | Week 0 | Random forest | Embedded + CAT score | 134 | 25 | 0.55 | 0.62 | 0.56 | 0.61 | 0.58 |
| Tier 1 | Clinical | Week 0 | Naïve Bayes | None | 47 | 47 | 0.57 | 0.58 | 0.54 | 0.61 | 0.58 |
| Tier 1 | Clinical | Week 0 | Elastic net | Embedded | 47 | 47 | 0.26 | 0.78 | 0.50 | 0.55 | 0.52 |
| Tier 1 | Clinical | Week 0 | SVM | None | 47 | 47 | 0.49 | 0.64 | 0.55 | 0.59 | 0.57 |
| Tier 1 | Clinical | Week 0 | GBM | Embedded | 47 | 47 | 0.45 | 0.58 | 0.48 | 0.55 | 0.51 |
| Tier 1 | Clinical | Week 0 | Random forest | Embedded | 47 | 47 | 0.40 | 0.67 | 0.52 | 0.56 | 0.54 |
| Tier 1 | Clinical | Week 0 | Naïve Bayes | CAT score | 47 | 25 | 0.56 | 0.60 | 0.55 | 0.61 | 0.58 |
| Tier 1 | Clinical | Week 0 | Elastic net | Embedded + CAT score | 47 | 25 | 0.43 | 0.66 | 0.53 | 0.57 | 0.54 |
| Tier 1 | Clinical | Week 0 | SVM | CAT score | 47 | 25 | 0.50 | 0.61 | 0.53 | 0.59 | 0.56 |
| Tier 1 | Clinical | Week 0 | GBM | Embedded + CAT score | 47 | 25 | 0.43 | 0.58 | 0.47 | 0.54 | 0.51 |
| Tier 1 | Clinical | Week 0 | Random forest | Embedded + CAT score | 47 | 25 | 0.41 | 0.63 | 0.49 | 0.55 | 0.52 |
| Tier 1 | Molecular | Week 0 | Naïve Bayes | None | 31 | 31 | 0.61 | 0.43 | 0.50 | 0.54 | 0.52 |
| Tier 1 | Molecular | Week 0 | Elastic net | Embedded | 31 | 31 | 0.38 | 0.71 | 0.54 | 0.57 | 0.55 |
| Tier 1 | Molecular | Week 0 | SVM | None | 31 | 31 | 0.49 | 0.73 | 0.62 | 0.62 | 0.61 |
| Tier 1 | Molecular | Week 0 | GBM | Embedded | 31 | 31 | 0.46 | 0.59 | 0.49 | 0.55 | 0.52 |
| Tier 1 | Molecular | Week 0 | Random forest | Embedded | 31 | 31 | 0.46 | 0.59 | 0.50 | 0.55 | 0.53 |
| Tier 1 | Molecular | Week 0 | Naïve Bayes | CAT score | 31 | 25 | 0.74 | 0.30 | 0.48 | 0.53 | 0.52 |
| Tier 1 | Molecular | Week 0 | Elastic net | Embedded + CAT score | 31 | 25 | 0.43 | 0.67 | 0.53 | 0.57 | 0.55 |
| Tier 1 | Molecular | Week 0 | SVM | CAT score | 31 | 25 | 0.48 | 0.74 | 0.61 | 0.62 | 0.61 |
| Tier 1 | Molecular | Week 0 | GBM | Embedded + CAT score | 31 | 25 | 0.45 | 0.59 | 0.49 | 0.55 | 0.52 |
| Tier 1 | Molecular | Week 0 | Random forest | Embedded + CAT score | 31 | 25 | 0.47 | 0.59 | 0.50 | 0.56 | 0.53 |
| Tier 1 | Neuroimaging | Week 0 | Naïve Bayes | None | 56 | 56 | 0.53 | 0.61 | 0.55 | 0.60 | 0.57 |
| Tier 1 | Neuroimaging | Week 0 | Elastic net | Embedded | 56 | 56 | 0.04 | 0.96 | 0.53 | 0.53 | 0.50 |
| Tier 1 | Neuroimaging | Week 0 | SVM | None | 56 | 56 | 0.36 | 0.72 | 0.54 | 0.56 | 0.54 |
| Tier 1 | Neuroimaging | Week 0 | GBM | Embedded | 56 | 56 | 0.47 | 0.60 | 0.51 | 0.56 | 0.53 |
| Tier 1 | Neuroimaging | Week 0 | Random forest | Embedded | 56 | 56 | 0.41 | 0.72 | 0.57 | 0.58 | 0.57 |
| Tier 1 | Neuroimaging | Week 0 | Naïve Bayes | CAT score | 56 | 25 | 0.49 | 0.58 | 0.51 | 0.57 | 0.54 |
| Tier 1 | Neuroimaging | Week 0 | Elastic net | Embedded + CAT score | 56 | 25 | 0.42 | 0.60 | 0.48 | 0.54 | 0.51 |
| Tier 1 | Neuroimaging | Week 0 | SVM | CAT score | 56 | 25 | 0.42 | 0.66 | 0.52 | 0.56 | 0.54 |
| Tier 1 | Neuroimaging | Week 0 | GBM | Embedded + CAT score | 56 | 25 | 0.44 | 0.61 | 0.50 | 0.55 | 0.52 |
| Tier 1 | Neuroimaging | Week 0 | Random forest | Embedded + CAT score | 56 | 25 | 0.43 | 0.69 | 0.55 | 0.58 | 0.56 |
| Tier 1 | Clinical+Neuroimaging | Week 0 | Naïve Bayes | None | 103 | 103 | 0.57 | 0.63 | 0.58 | 0.62 | 0.60 |
| Tier 1 | Clinical+Neuroimaging | Week 0 | Elastic net | Embedded | 103 | 103 | 0.12 | 0.91 | 0.56 | 0.54 | 0.52 |
| Tier 1 | Clinical+Neuroimaging | Week 0 | SVM | None | 103 | 103 | 0.44 | 0.76 | 0.62 | 0.60 | 0.60 |
| Tier 1 | Clinical+Neuroimaging | Week 0 | GBM | Embedded | 103 | 103 | 0.48 | 0.60 | 0.52 | 0.57 | 0.54 |
| Tier 1 | Clinical+Neuroimaging | Week 0 | Random forest | Embedded | 103 | 103 | 0.41 | 0.76 | 0.61 | 0.59 | 0.59 |
| Tier 1 | Clinical+Neuroimaging | Week 0 | Naïve Bayes | CAT score | 103 | 25 | 0.55 | 0.59 | 0.55 | 0.60 | 0.57 |
| Tier 1 | Clinical+Neuroimaging | Week 0 | Elastic net | Embedded + CAT score | 103 | 25 | 0.49 | 0.63 | 0.54 | 0.58 | 0.56 |
| Tier 1 | Clinical+Neuroimaging | Week 0 | SVM | CAT score | 103 | 25 | 0.49 | 0.63 | 0.54 | 0.59 | 0.56 |
| Tier 1 | Clinical+Neuroimaging | Week 0 | GBM | Embedded + CAT score | 103 | 25 | 0.46 | 0.54 | 0.47 | 0.54 | 0.50 |
| Tier 1 | Clinical+Neuroimaging | Week 0 | Random forest | Embedded + CAT score | 103 | 25 | 0.45 | 0.64 | 0.52 | 0.57 | 0.54 |
| Tier 1 | Neuroimaging+Molecular | Week 0 | Naïve Bayes | None | 87 | 87 | 0.60 | 0.51 | 0.53 | 0.60 | 0.56 |
| Tier 1 | Neuroimaging+Molecular | Week 0 | Elastic net | Embedded | 87 | 87 | 0.23 | 0.79 | 0.49 | 0.54 | 0.51 |
| Tier 1 | Neuroimaging+Molecular | Week 0 | SVM | None | 87 | 87 | 0.44 | 0.70 | 0.56 | 0.58 | 0.57 |
| Tier 1 | Neuroimaging+Molecular | Week 0 | GBM | Embedded | 87 | 87 | 0.45 | 0.56 | 0.48 | 0.54 | 0.51 |
| Tier 1 | Neuroimaging+Molecular | Week 0 | Random forest | Embedded | 87 | 87 | 0.39 | 0.70 | 0.53 | 0.56 | 0.54 |
| Tier 1 | Neuroimaging+Molecular | Week 0 | Naïve Bayes | CAT score | 87 | 25 | 0.61 | 0.44 | 0.52 | 0.56 | 0.53 |
| Tier 1 | Neuroimaging+Molecular | Week 0 | Elastic net | Embedded + CAT score | 87 | 25 | 0.49 | 0.62 | 0.53 | 0.58 | 0.55 |
| Tier 1 | Neuroimaging+Molecular | Week 0 | SVM | CAT score | 87 | 25 | 0.50 | 0.66 | 0.57 | 0.60 | 0.58 |
| Tier 1 | Neuroimaging+Molecular | Week 0 | GBM | Embedded + CAT score | 87 | 25 | 0.44 | 0.59 | 0.49 | 0.55 | 0.52 |
| Tier 1 | Neuroimaging+Molecular | Week 0 | Random forest | Embedded + CAT score | 87 | 25 | 0.45 | 0.64 | 0.52 | 0.57 | 0.54 |
| Tier 1 | Clinical+Molecular | Week 0 | Naïve Bayes | None | 78 | 78 | 0.58 | 0.52 | 0.53 | 0.57 | 0.55 |
| Tier 1 | Clinical+Molecular | Week 0 | Elastic net | Embedded | 78 | 78 | 0.34 | 0.75 | 0.55 | 0.57 | 0.55 |
| Tier 1 | Clinical+Molecular | Week 0 | SVM | None | 78 | 78 | 0.47 | 0.71 | 0.59 | 0.60 | 0.59 |
| Tier 1 | Clinical+Molecular | Week 0 | GBM | Embedded | 78 | 78 | 0.51 | 0.59 | 0.52 | 0.58 | 0.55 |
| Tier 1 | Clinical+Molecular | Week 0 | Random forest | Embedded | 78 | 78 | 0.45 | 0.66 | 0.54 | 0.58 | 0.56 |
| Tier 1 | Clinical+Molecular | Week 0 | Naïve Bayes | CAT score | 78 | 25 | 0.75 | 0.38 | 0.52 | 0.63 | 0.56 |
| Tier 1 | Clinical+Molecular | Week 0 | Elastic net | Embedded + CAT score | 78 | 25 | 0.55 | 0.60 | 0.54 | 0.61 | 0.57 |
| Tier 1 | Clinical+Molecular | Week 0 | SVM | CAT score | 78 | 25 | 0.52 | 0.62 | 0.55 | 0.60 | 0.57 |
| Tier 1 | Clinical+Molecular | Week 0 | GBM | Embedded + CAT score | 78 | 25 | 0.54 | 0.56 | 0.51 | 0.59 | 0.55 |
| Tier 1 | Clinical+Molecular | Week 0 | Random forest | Embedded + CAT score | 78 | 25 | 0.52 | 0.60 | 0.53 | 0.59 | 0.56 |
| Tier 1 | All | Week 0 + week 2 | Naïve Bayes | None | 214 | 214 | 0.65 | 0.56 | 0.57 | 0.65 | 0.61 |
| Tier 1 | All | Week 0 + week 2 | Elastic net | Embedded | 214 | 214 | 0.48 | 0.81 | 0.71 | 0.64 | 0.65 |
| Tier 1 | All | Week 0 + week 2 | SVM | None | 214 | 214 | 0.39 | 0.83 | 0.69 | 0.62 | 0.61 |
| Tier 1 | All | Week 0 + week 2 | GBM | Embedded | 214 | 214 | 0.53 | 0.66 | 0.59 | 0.61 | 0.60 |
| Tier 1 | All | Week 0 + week 2 | Random forest | Embedded | 214 | 214 | 0.59 | 0.73 | 0.67 | 0.67 | 0.66 |
| Tier 1 | All | Week 0 + week 2 | Naïve Bayes | CAT score | 214 | 25 | 0.61 | 0.55 | 0.65 | 0.64 | 0.58 |
| Tier 1 | All | Week 0 + week 2 | Elastic net | Embedded + CAT score | 214 | 25 | 0.56 | 0.73 | 0.65 | 0.65 | 0.64 |
| Tier 1 | All | Week 0 + week 2 | SVM | CAT score | 214 | 25 | 0.54 | 0.73 | 0.64 | 0.64 | 0.63 |
| Tier 1 | All | Week 0 + week 2 | GBM | Embedded + CAT score | 214 | 25 | 0.49 | 0.65 | 0.56 | 0.59 | 0.57 |
| Tier 1 | All | Week 0 + week 2 | Random forest | Embedded + CAT score | 214 | 25 | 0.54 | 0.72 | 0.63 | 0.64 | 0.63 |
| Tier 1 | Clinical | Week 0 + week 2 | Naïve Bayes | None | 55 | 55 | 0.69 | 0.63 | 0.63 | 0.70 | 0.66 |
| Tier 1 | Clinical | Week 0 + week 2 | Elastic net | Embedded | 55 | 55 | 0.52 | 0.80 | 0.71 | 0.65 | 0.66 |
| Tier 1 | Clinical | Week 0 + week 2 | SVM | None | 55 | 55 | 0.57 | 0.73 | 0.67 | 0.66 | 0.65 |
| Tier 1 | Clinical | Week 0 + week 2 | GBM | Embedded | 55 | 55 | 0.54 | 0.65 | 0.59 | 0.61 | 0.60 |
| Tier 1 | Clinical | Week 0 + week 2 | Random forest | Embedded | 55 | 55 | 0.59 | 0.70 | 0.65 | 0.66 | 0.65 |
| Tier 1 | Clinical | Week 0 + week 2 | Naïve Bayes | CAT score | 55 | 25 | 0.65 | 0.65 | 0.62 | 0.68 | 0.65 |
| Tier 1 | Clinical | Week 0 + week 2 | Elastic net | Embedded + CAT score | 55 | 25 | 0.54 | 0.71 | 0.63 | 0.64 | 0.62 |
| Tier 1 | Clinical | Week 0 + week 2 | SVM | CAT score | 55 | 25 | 0.52 | 0.75 | 0.65 | 0.63 | 0.63 |
| Tier 1 | Clinical | Week 0 + week 2 | GBM | Embedded + CAT score | 55 | 25 | 0.51 | 0.63 | 0.56 | 0.60 | 0.57 |
| Tier 1 | Clinical | Week 0 + week 2 | Random forest | Embedded + CAT score | 55 | 25 | 0.55 | 0.73 | 0.65 | 0.65 | 0.64 |
| Tier 1 | Molecular | Week 0 + week 2 | Naïve Bayes | None | 52 | 52 | 0.74 | 0.38 | 0.53 | 0.62 | 0.56 |
| Tier 1 | Molecular | Week 0 + week 2 | Elastic net | Embedded | 52 | 52 | 0.40 | 0.70 | 0.55 | 0.57 | 0.55 |
| Tier 1 | Molecular | Week 0 + week 2 | SVM | None | 52 | 52 | 0.35 | 0.84 | 0.67 | 0.61 | 0.60 |
| Tier 1 | Molecular | Week 0 + week 2 | GBM | Embedded | 52 | 52 | 0.41 | 0.65 | 0.51 | 0.55 | 0.53 |
| Tier 1 | Molecular | Week 0 + week 2 | Random forest | Embedded | 52 | 52 | 0.45 | 0.64 | 0.54 | 0.57 | 0.55 |
| Tier 1 | Molecular | Week 0 + week 2 | Naïve Bayes | CAT score | 52 | 25 | 0.76 | 0.33 | 0.51 | 0.61 | 0.55 |
| Tier 1 | Molecular | Week 0 + week 2 | Elastic net | Embedded + CAT score | 52 | 25 | 0.46 | 0.63 | 0.53 | 0.56 | 0.54 |
| Tier 1 | Molecular | Week 0 + week 2 | SVM | CAT score | 52 | 25 | 0.44 | 0.69 | 0.56 | 0.58 | 0.56 |
| Tier 1 | Molecular | Week 0 + week 2 | GBM | Embedded + CAT score | 52 | 25 | 0.43 | 0.65 | 0.52 | 0.56 | 0.54 |
| Tier 1 | Molecular | Week 0 + week 2 | Random forest | Embedded + CAT score | 52 | 25 | 0.45 | 0.63 | 0.53 | 0.56 | 0.54 |
| Tier 1 | Neuroimaging | Week 0 + week 2 | Naïve Bayes | None | 107 | 107 | 0.57 | 0.59 | 0.55 | 0.60 | 0.58 |
| Tier 1 | Neuroimaging | Week 0 + week 2 | Elastic net | Embedded | 107 | 107 | 0.07 | 0.93 | 0.49 | 0.53 | 0.50 |
| Tier 1 | Neuroimaging | Week 0 + week 2 | SVM | None | 107 | 107 | 0.37 | 0.70 | 0.52 | 0.55 | 0.53 |
| Tier 1 | Neuroimaging | Week 0 + week 2 | GBM | Embedded | 107 | 107 | 0.47 | 0.56 | 0.49 | 0.54 | 0.51 |
| Tier 1 | Neuroimaging | Week 0 + week 2 | Random forest | Embedded | 107 | 107 | 0.41 | 0.70 | 0.55 | 0.57 | 0.55 |
| Tier 1 | Neuroimaging | Week 0 + week 2 | Naïve Bayes | CAT score | 107 | 25 | 0.51 | 0.58 | 0.52 | 0.57 | 0.54 |
| Tier 1 | Neuroimaging | Week 0 + week 2 | Elastic net | Embedded + CAT score | 107 | 25 | 0.41 | 0.56 | 0.45 | 0.51 | 0.49 |
| Tier 1 | Neuroimaging | Week 0 + week 2 | SVM | CAT score | 107 | 25 | 0.40 | 0.60 | 0.48 | 0.53 | 0.50 |
| Tier 1 | Neuroimaging | Week 0 + week 2 | GBM | Embedded + CAT score | 107 | 25 | 0.45 | 0.53 | 0.46 | 0.52 | 0.49 |
| Tier 1 | Neuroimaging | Week 0 + week 2 | Random forest | Embedded + CAT score | 107 | 25 | 0.43 | 0.61 | 0.50 | 0.55 | 0.52 |
| Tier 1 | Clinical+Neuroimaging | Week 0 + week 2 | Naïve Bayes | None | 162 | 162 | 0.61 | 0.63 | 0.60 | 0.64 | 0.62 |
| Tier 1 | Clinical+Neuroimaging | Week 0 + week 2 | Elastic net | Embedded | 162 | 162 | 0.46 | 0.83 | 0.71 | 0.64 | 0.65 |
| Tier 1 | Clinical+Neuroimaging | Week 0 + week 2 | SVM | None | 162 | 162 | 0.54 | 0.75 | 0.67 | 0.65 | 0.65 |
| Tier 1 | Clinical+Neuroimaging | Week 0 + week 2 | GBM | Embedded | 162 | 162 | 0.55 | 0.65 | 0.59 | 0.62 | 0.60 |
| Tier 1 | Clinical+Neuroimaging | Week 0 + week 2 | Random forest | Embedded | 162 | 162 | 0.59 | 0.73 | 0.67 | 0.67 | 0.66 |
| Tier 1 | Clinical+Neuroimaging | Week 0 + week 2 | Naïve Bayes | CAT score | 162 | 25 | 0.63 | 0.66 | 0.62 | 0.66 | 0.64 |
| Tier 1 | Clinical+Neuroimaging | Week 0 + week 2 | Elastic net | Embedded + CAT score | 162 | 25 | 0.55 | 0.66 | 0.59 | 0.62 | 0.60 |
| Tier 1 | Clinical+Neuroimaging | Week 0 + week 2 | SVM | CAT score | 162 | 25 | 0.52 | 0.68 | 0.60 | 0.61 | 0.60 |
| Tier 1 | Clinical+Neuroimaging | Week 0 + week 2 | GBM | Embedded + CAT score | 162 | 25 | 0.49 | 0.62 | 0.53 | 0.58 | 0.55 |
| Tier 1 | Clinical+Neuroimaging | Week 0 + week 2 | Random forest | Embedded + CAT score | 162 | 25 | 0.56 | 0.70 | 0.62 | 0.64 | 0.63 |
| Tier 1 | Neuroimaging+Molecular | Week 0 + week 2 | Naïve Bayes | None | 159 | 159 | 0.65 | 0.48 | 0.53 | 0.62 | 0.57 |
| Tier 1 | Neuroimaging+Molecular | Week 0 + week 2 | Elastic net | Embedded | 159 | 159 | 0.30 | 0.74 | 0.51 | 0.55 | 0.52 |
| Tier 1 | Neuroimaging+Molecular | Week 0 + week 2 | SVM | None | 159 | 159 | 0.32 | 0.76 | 0.55 | 0.56 | 0.54 |
| Tier 1 | Neuroimaging+Molecular | Week 0 + week 2 | GBM | Embedded | 159 | 159 | 0.48 | 0.59 | 0.51 | 0.56 | 0.53 |
| Tier 1 | Neuroimaging+Molecular | Week 0 + week 2 | Random forest | Embedded | 159 | 159 | 0.43 | 0.64 | 0.53 | 0.56 | 0.54 |
| Tier 1 | Neuroimaging+Molecular | Week 0 + week 2 | Naïve Bayes | CAT score | 159 | 25 | 0.44 | 0.63 | 0.57 | 0.56 | 0.53 |
| Tier 1 | Neuroimaging+Molecular | Week 0 + week 2 | Elastic net | Embedded + CAT score | 159 | 25 | 0.45 | 0.60 | 0.5 | 0.55 | 0.52 |
| Tier 1 | Neuroimaging+Molecular | Week 0 + week 2 | SVM | CAT score | 159 | 25 | 0.47 | 0.60 | 0.52 | 0.56 | 0.54 |
| Tier 1 | Neuroimaging+Molecular | Week 0 + week 2 | GBM | Embedded + CAT score | 159 | 25 | 0.44 | 0.58 | 0.49 | 0.54 | 0.51 |
| Tier 1 | Neuroimaging+Molecular | Week 0 + week 2 | Random forest | Embedded + CAT score | 159 | 25 | 0.45 | 0.61 | 0.51 | 0.55 | 0.53 |
| Tier 1 | Clinical+Molecular | Week 0 + week 2 | Naïve Bayes | None | 107 | 107 | 0.73 | 0.51 | 0.58 | 0.68 | 0.62 |
| Tier 1 | Clinical+Molecular | Week 0 + week 2 | Elastic net | Embedded | 107 | 107 | 0.51 | 0.81 | 0.71 | 0.65 | 0.66 |
| Tier 1 | Clinical+Molecular | Week 0 + week 2 | SVM | None | 107 | 107 | 0.41 | 0.84 | 0.71 | 0.63 | 0.62 |
| Tier 1 | Clinical+Molecular | Week 0 + week 2 | GBM | Embedded | 107 | 107 | 0.52 | 0.70 | 0.61 | 0.62 | 0.61 |
| Tier 1 | Clinical+Molecular | Week 0 + week 2 | Random forest | Embedded | 107 | 107 | 0.56 | 0.69 | 0.62 | 0.64 | 0.63 |
| Tier 1 | Clinical+Molecular | Week 0 + week 2 | Naïve Bayes | CAT score | 107 | 25 | 0.74 | 0.52 | 0.59 | 0.71 | 0.63 |
| Tier 1 | Clinical+Molecular | Week 0 + week 2 | Elastic net | Embedded + CAT score | 107 | 25 | 0.57 | 0.75 | 0.67 | 0.66 | 0.66 |
| Tier 1 | Clinical+Molecular | Week 0 + week 2 | SVM | CAT score | 107 | 25 | 0.55 | 0.73 | 0.65 | 0.65 | 0.64 |
| Tier 1 | Clinical+Molecular | Week 0 + week 2 | GBM | Embedded + CAT score | 107 | 25 | 0.52 | 0.67 | 0.59 | 0.61 | 0.60 |
| Tier 1 | Clinical+Molecular | Week 0 + week 2 | Random forest | Embedded + CAT score | 107 | 25 | 0.57 | 0.71 | 0.64 | 0.65 | 0.64 |
| Tier 2 | All | Week 0 | Naïve Bayes | None | 1152 | 1152 | 0.50 | 0.62 | 0.54 | 0.60 | 0.56 |
| Tier 2 | All | Week 0 | Elastic net | Embedded | 1152 | 1152 | 0.28 | 0.78 | 0.53 | 0.56 | 0.53 |
| Tier 2 | All | Week 0 | SVM | None | 1152 | 1152 | 0.22 | 0.88 | 0.63 | 0.56 | 0.55 |
| Tier 2 | All | Week 0 | GBM | Embedded | 1152 | 1152 | 0.42 | 0.63 | 0.49 | 0.55 | 0.52 |
| Tier 2 | All | Week 0 | Random forest | Embedded | 1152 | 1152 | 0.24 | 0.82 | 0.55 | 0.55 | 0.53 |
| Tier 2 | All | Week 0 | Naïve Bayes | CAT score | 1152 | 25 | 0.67 | 0.43 | 0.52 | 0.58 | 0.55 |
| Tier 2 | All | Week 0 | Elastic net | Embedded + CAT score | 1152 | 25 | 0.51 | 0.58 | 0.52 | 0.57 | 0.55 |
| Tier 2 | All | Week 0 | SVM | CAT score | 1152 | 25 | 0.52 | 0.60 | 0.54 | 0.59 | 0.56 |
| Tier 2 | All | Week 0 | GBM | Embedded + CAT score | 1152 | 25 | 0.50 | 0.53 | 0.48 | 0.55 | 0.51 |
| Tier 2 | All | Week 0 | Random forest | Embedded + CAT score | 1152 | 25 | 0.52 | 0.59 | 0.53 | 0.59 | 0.56 |
| Tier 2 | Clinical | Week 0 | Naïve Bayes | None | 194 | 194 | 0.65 | 0.45 | 0.52 | 0.64 | 0.55 |
| Tier 2 | Clinical | Week 0 | Elastic net | Embedded | 194 | 194 | 0.28 | 0.77 | 0.53 | 0.55 | 0.53 |
| Tier 2 | Clinical | Week 0 | SVM | None | 194 | 194 | 0.39 | 0.73 | 0.57 | 0.58 | 0.56 |
| Tier 2 | Clinical | Week 0 | GBM | Embedded | 194 | 194 | 0.45 | 0.58 | 0.49 | 0.55 | 0.52 |
| Tier 2 | Clinical | Week 0 | Random forest | Embedded | 194 | 194 | 0.41 | 0.69 | 0.55 | 0.57 | 0.55 |
| Tier 2 | Clinical | Week 0 | Naïve Bayes | CAT score | 194 | 25 | 0.68 | 0.41 | 0.51 | 0.62 | 0.55 |
| Tier 2 | Clinical | Week 0 | Elastic net | Embedded + CAT score | 194 | 25 | 0.52 | 0.58 | 0.52 | 0.58 | 0.55 |
| Tier 2 | Clinical | Week 0 | SVM | CAT score | 194 | 25 | 0.51 | 0.60 | 0.53 | 0.58 | 0.56 |
| Tier 2 | Clinical | Week 0 | GBM | Embedded + CAT score | 194 | 25 | 0.53 | 0.57 | 0.52 | 0.58 | 0.55 |
| Tier 2 | Clinical | Week 0 | Random forest | Embedded + CAT score | 194 | 25 | 0.51 | 0.62 | 0.54 | 0.59 | 0.57 |
| Tier 2 | Molecular | Week 0 | Naïve Bayes | None | 733 | 733 | 0.43 | 0.56 | 0.46 | 0.53 | 0.49 |
| Tier 2 | Molecular | Week 0 | Elastic net | Embedded | 733 | 733 | 0.28 | 0.77 | 0.51 | 0.55 | 0.52 |
| Tier 2 | Molecular | Week 0 | SVM | None | 733 | 733 | 0.16 | 0.87 | 0.49 | 0.54 | 0.52 |
| Tier 2 | Molecular | Week 0 | GBM | Embedded | 733 | 733 | 0.39 | 0.61 | 0.47 | 0.53 | 0.50 |
| Tier 2 | Molecular | Week 0 | Random forest | Embedded | 733 | 733 | 0.19 | 0.80 | 0.45 | 0.53 | 0.50 |
| Tier 2 | Molecular | Week 0 | Naïve Bayes | CAT score | 733 | 25 | 0.70 | 0.34 | 0.49 | 0.56 | 0.52 |
| Tier 2 | Molecular | Week 0 | Elastic net | Embedded + CAT score | 733 | 25 | 0.48 | 0.54 | 0.48 | 0.55 | 0.51 |
| Tier 2 | Molecular | Week 0 | SVM | CAT score | 733 | 25 | 0.45 | 0.59 | 0.50 | 0.55 | 0.52 |
| Tier 2 | Molecular | Week 0 | GBM | Embedded + CAT score | 733 | 25 | 0.43 | 0.53 | 0.44 | 0.52 | 0.48 |
| Tier 2 | Molecular | Week 0 | Random forest | Embedded + CAT score | 733 | 25 | 0.46 | 0.55 | 0.48 | 0.53 | 0.50 |
| Tier 2 | Neuroimaging | Week 0 | Naïve Bayes | None | 225 | 225 | 0.53 | 0.50 | 0.48 | 0.55 | 0.51 |
| Tier 2 | Neuroimaging | Week 0 | Elastic net | Embedded | 225 | 225 | 0.07 | 0.93 | 0.49 | 0.53 | 0.50 |
| Tier 2 | Neuroimaging | Week 0 | SVM | None | 225 | 225 | 0.39 | 0.67 | 0.51 | 0.56 | 0.53 |
| Tier 2 | Neuroimaging | Week 0 | GBM | Embedded | 225 | 225 | 0.49 | 0.60 | 0.52 | 0.57 | 0.55 |
| Tier 2 | Neuroimaging | Week 0 | Random forest | Embedded | 225 | 225 | 0.43 | 0.67 | 0.53 | 0.57 | 0.55 |
| Tier 2 | Neuroimaging | Week 0 | Naïve Bayes | CAT score | 225 | 25 | 0.52 | 0.57 | 0.52 | 0.58 | 0.55 |
| Tier 2 | Neuroimaging | Week 0 | Elastic net | Embedded + CAT score | 225 | 25 | 0.45 | 0.59 | 0.49 | 0.54 | 0.52 |
| Tier 2 | Neuroimaging | Week 0 | SVM | CAT score | 225 | 25 | 0.41 | 0.65 | 0.51 | 0.56 | 0.53 |
| Tier 2 | Neuroimaging | Week 0 | GBM | Embedded + CAT score | 225 | 25 | 0.44 | 0.60 | 0.49 | 0.55 | 0.52 |
| Tier 2 | Neuroimaging | Week 0 | Random forest | Embedded + CAT score | 225 | 25 | 0.41 | 0.67 | 0.52 | 0.56 | 0.54 |
| Tier 2 | Clinical+Neuroimaging | Week 0 | Naïve Bayes | None | 419 | 419 | 0.61 | 0.50 | 0.52 | 0.62 | 0.55 |
| Tier 2 | Clinical+Neuroimaging | Week 0 | Elastic net | Embedded | 419 | 419 | 0.16 | 0.86 | 0.50 | 0.54 | 0.51 |
| Tier 2 | Clinical+Neuroimaging | Week 0 | SVM | None | 419 | 419 | 0.40 | 0.74 | 0.58 | 0.59 | 0.57 |
| Tier 2 | Clinical+Neuroimaging | Week 0 | GBM | Embedded | 419 | 419 | 0.49 | 0.60 | 0.52 | 0.57 | 0.54 |
| Tier 2 | Clinical+Neuroimaging | Week 0 | Random forest | Embedded | 419 | 419 | 0.40 | 0.74 | 0.57 | 0.58 | 0.57 |
| Tier 2 | Clinical+Neuroimaging | Week 0 | Naïve Bayes | CAT score | 419 | 25 | 0.71 | 0.38 | 0.52 | 0.63 | 0.55 |
| Tier 2 | Clinical+Neuroimaging | Week 0 | Elastic net | Embedded + CAT score | 419 | 25 | 0.52 | 0.59 | 0.53 | 0.59 | 0.56 |
| Tier 2 | Clinical+Neuroimaging | Week 0 | SVM | CAT score | 419 | 25 | 0.49 | 0.62 | 0.53 | 0.58 | 0.55 |
| Tier 2 | Clinical+Neuroimaging | Week 0 | GBM | Embedded + CAT score | 419 | 25 | 0.53 | 0.58 | 0.53 | 0.58 | 0.56 |
| Tier 2 | Clinical+Neuroimaging | Week 0 | Random forest | Embedded + CAT score | 419 | 25 | 0.51 | 0.64 | 0.56 | 0.6 | 0.58 |
| Tier 2 | Neuroimaging+Molecular | Week 0 | Naïve Bayes | None | 958 | 958 | 0.44 | 0.59 | 0.49 | 0.55 | 0.52 |
| Tier 2 | Neuroimaging+Molecular | Week 0 | Elastic net | Embedded | 958 | 958 | 0.24 | 0.78 | 0.49 | 0.54 | 0.51 |
| Tier 2 | Neuroimaging+Molecular | Week 0 | SVM | None | 958 | 958 | 0.18 | 0.85 | 0.52 | 0.54 | 0.51 |
| Tier 2 | Neuroimaging+Molecular | Week 0 | GBM | Embedded | 958 | 958 | 0.41 | 0.62 | 0.48 | 0.55 | 0.52 |
| Tier 2 | Neuroimaging+Molecular | Week 0 | Random forest | Embedded | 958 | 958 | 0.22 | 0.80 | 0.48 | 0.54 | 0.51 |
| Tier 2 | Neuroimaging+Molecular | Week 0 | Naïve Bayes | CAT score | 958 | 25 | 0.61 | 0.49 | 0.52 | 0.58 | 0.55 |
| Tier 2 | Neuroimaging+Molecular | Week 0 | Elastic net | Embedded + CAT score | 958 | 25 | 0.48 | 0.59 | 0.51 | 0.56 | 0.53 |
| Tier 2 | Neuroimaging+Molecular | Week 0 | SVM | CAT score | 958 | 25 | 0.49 | 0.61 | 0.52 | 0.58 | 0.55 |
| Tier 2 | Neuroimaging+Molecular | Week 0 | GBM | Embedded + CAT score | 958 | 25 | 0.50 | 0.58 | 0.51 | 0.57 | 0.54 |
| Tier 2 | Neuroimaging+Molecular | Week 0 | Random forest | Embedded + CAT score | 958 | 25 | 0.46 | 0.63 | 0.52 | 0.57 | 0.54 |
| Tier 2 | Clinical+Molecular | Week 0 | Naïve Bayes | None | 927 | 927 | 0.53 | 0.58 | 0.53 | 0.59 | 0.56 |
| Tier 2 | Clinical+Molecular | Week 0 | Elastic net | Embedded | 927 | 927 | 0.35 | 0.76 | 0.56 | 0.57 | 0.55 |
| Tier 2 | Clinical+Molecular | Week 0 | SVM | None | 927 | 927 | 0.19 | 0.90 | 0.63 | 0.56 | 0.54 |
| Tier 2 | Clinical+Molecular | Week 0 | GBM | Embedded | 927 | 927 | 0.39 | 0.62 | 0.47 | 0.54 | 0.50 |
| Tier 2 | Clinical+Molecular | Week 0 | Random forest | Embedded | 927 | 927 | 0.23 | 0.81 | 0.52 | 0.54 | 0.52 |
| Tier 2 | Clinical+Molecular | Week 0 | Naïve Bayes | CAT score | 927 | 25 | 0.63 | 0.49 | 0.53 | 0.6 | 0.56 |
| Tier 2 | Clinical+Molecular | Week 0 | Elastic net | Embedded + CAT score | 927 | 25 | 0.50 | 0.59 | 0.52 | 0.58 | 0.55 |
| Tier 2 | Clinical+Molecular | Week 0 | SVM | CAT score | 927 | 25 | 0.51 | 0.61 | 0.53 | 0.59 | 0.56 |
| Tier 2 | Clinical+Molecular | Week 0 | GBM | Embedded + CAT score | 927 | 25 | 0.48 | 0.55 | 0.48 | 0.55 | 0.51 |
| Tier 2 | Clinical+Molecular | Week 0 | Random forest | Embedded + CAT score | 927 | 25 | 0.52 | 0.60 | 0.53 | 0.59 | 0.56 |

**Figure S1**: Flow diagram of CAN-BIND-1 participants

**
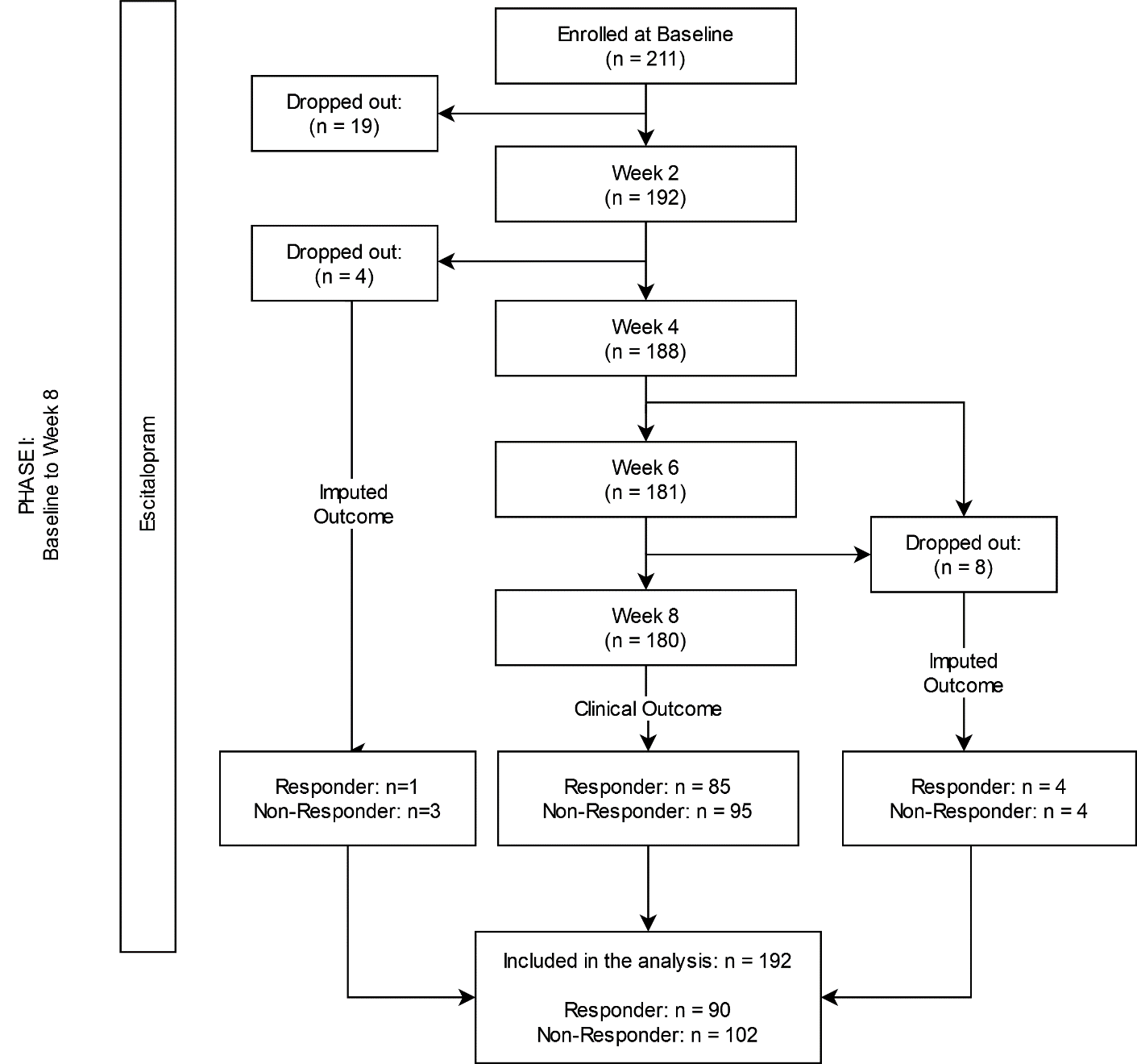
**

**Figure S2**: Visualization of missing values for tier 1 (week 0) (A-C); tier 2 (week 0) (D-F), and tier 1 (week 2) (G-I) data per variable and subject ID.


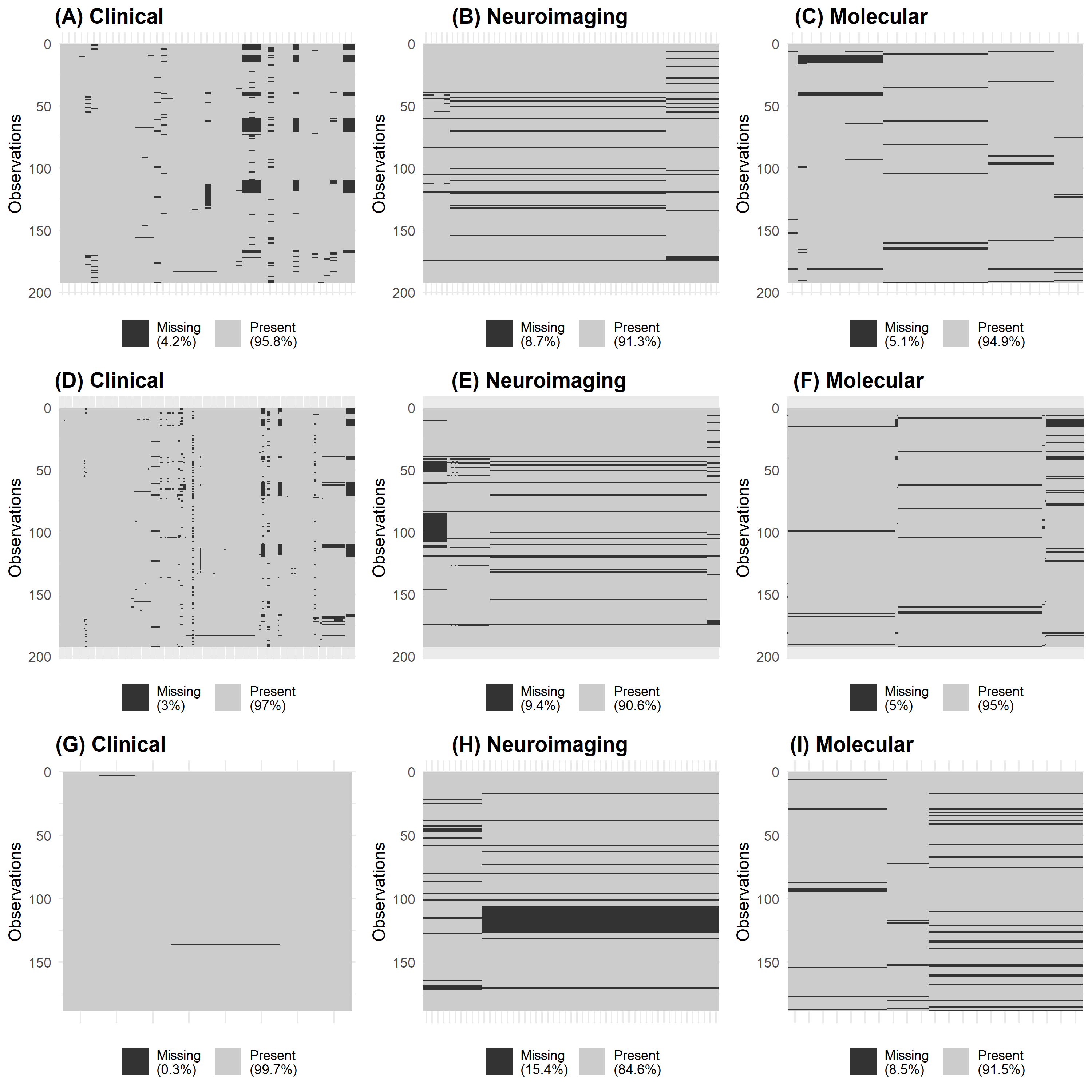


**Figure S3:** Variable importance extracted from CAT score and embedded elastic net, gradient boosting, and random forest models for all of the variables in tier 1 (week 0) (A), tier 2 (week 0) (B), and tier 1 (week 0 + week 2) (C).

**Figure S4:** Variable importance based on the scores extracted from CAT score and embedded elastic net, gradient boosting, and random forest models for the variables of each modality in tier 1 (week 0) (A-C), and the combination of two modalities in tier 1 (week 0) (D-F).


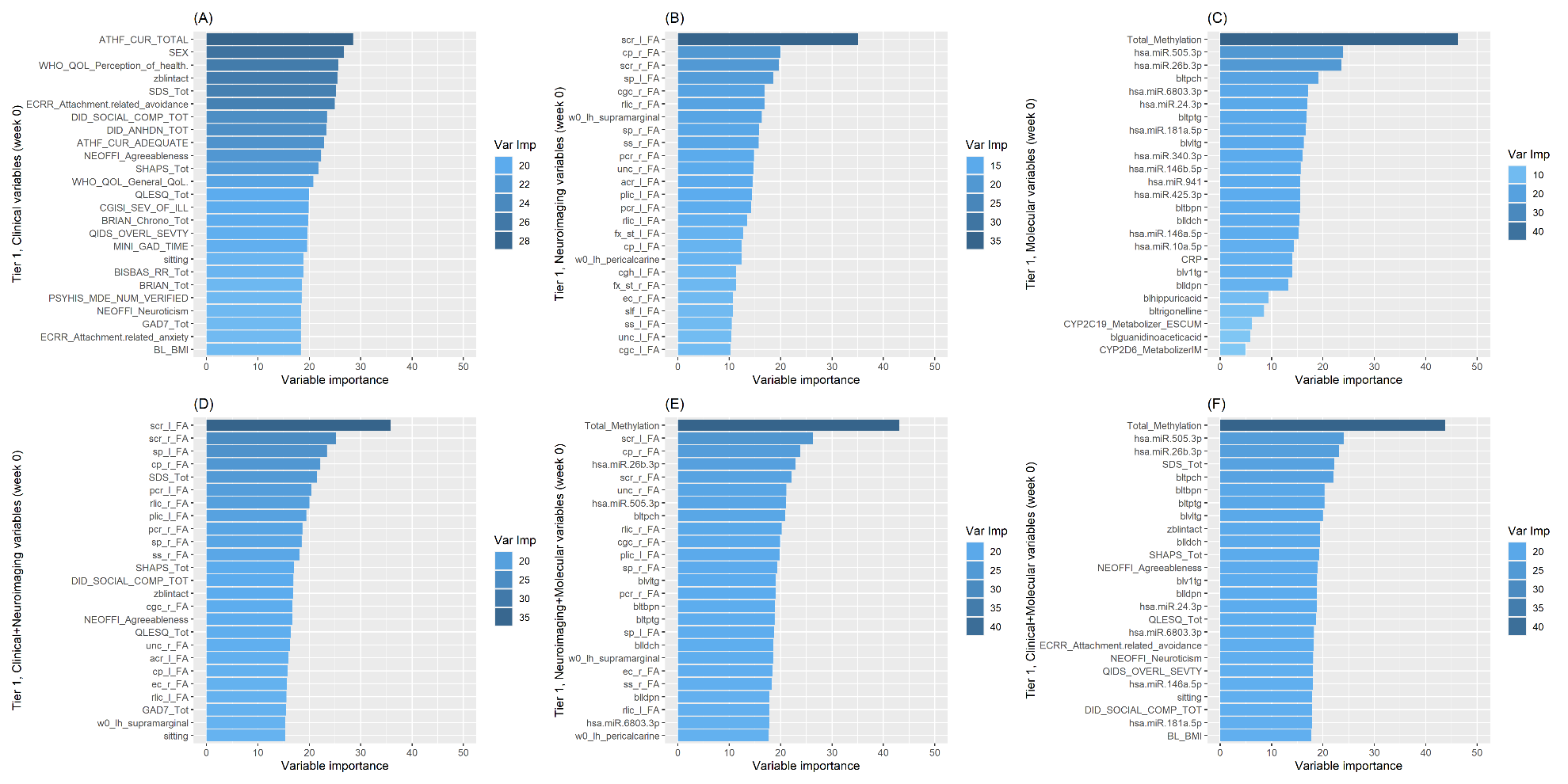


**Figure S5:** Variable importance based on the scores extracted from CAT score and embedded elastic net, gradient boosting, and random forest models for the variables of each modality in tier 2 (week 0) (A-C), and the combination of two modalities in tier 2 (week 0).


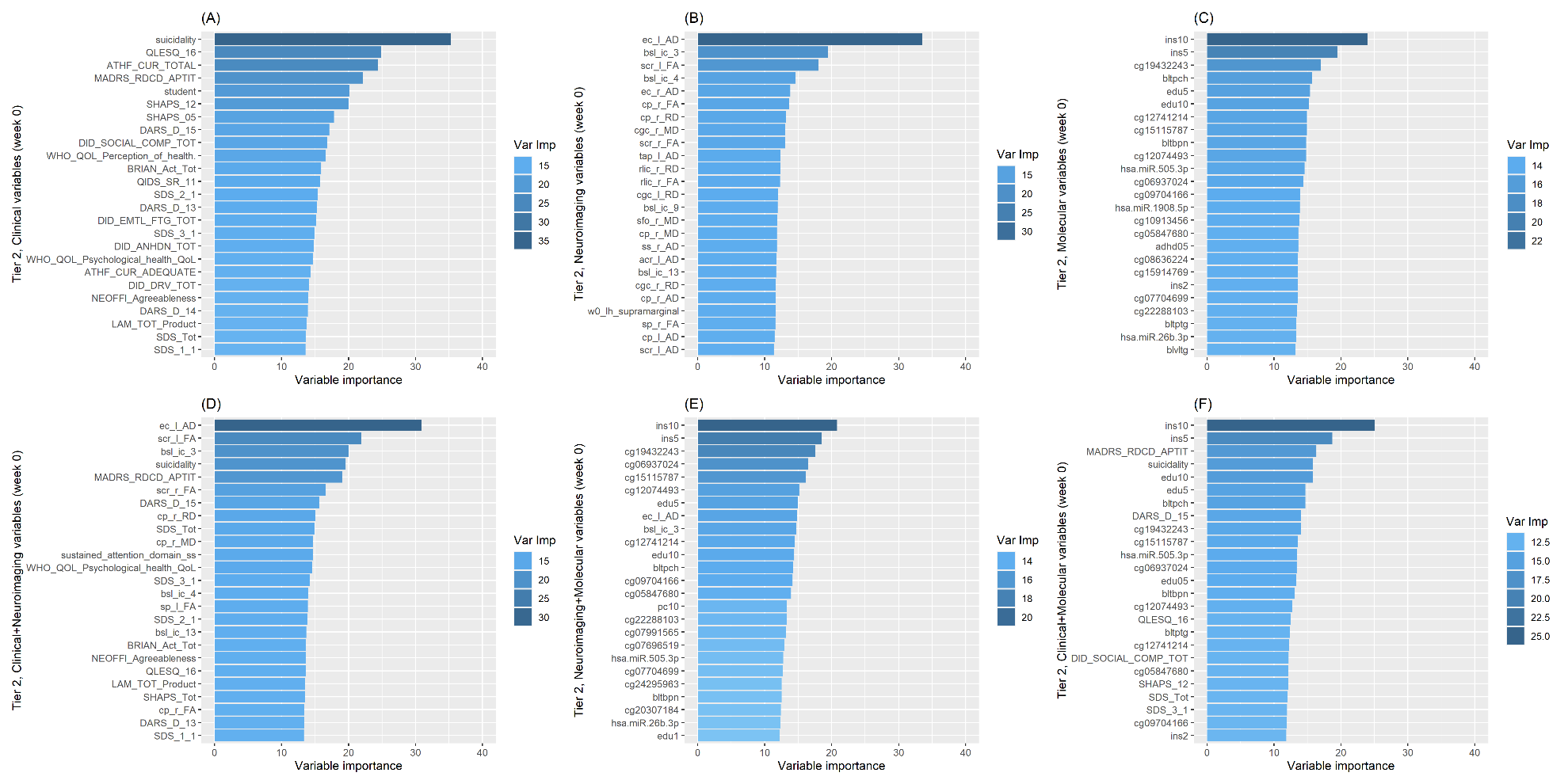


**Figure S6:** Variable importance based on the scores extracted from CAT score and embedded elastic net, gradient boosting, and random forest models for the variables of each modality in tier 1 (week 0 + week 2) (A-C), and the combination of two modalities in tier 1 (week 0 + week 2).


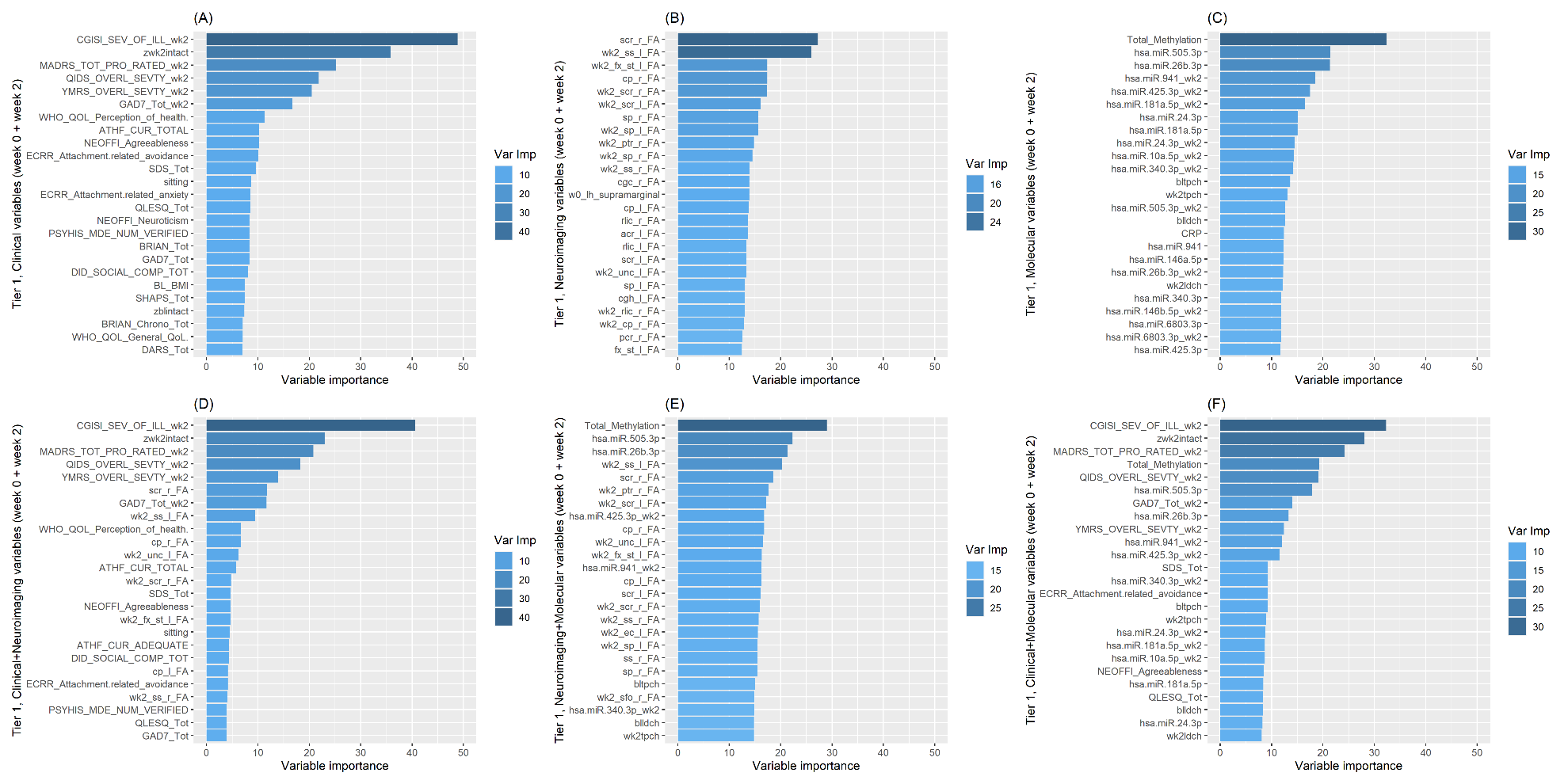


**Figure S7: Receiver Operating Characteristic (ROC) curves for tier 1 week 0 predictor models.** The most predictive (highest AUC) model was chosen for each predictor modality and combination: (A) clinical data, Naive Bayes model with feature selection (B) molecular data, SVM model w/o feature selection (C) neuroimaging data, Naive Bayes model w/o feature selection (D) clinical + neuroimaging, Naive Bayes model w/o feature selection (E) neuroimaging + molecular, SVM model with feature selection (F) clinical + molecular, SVM model w/o feature selection (G) clinical + neuroimaging + molecular, SVM model w/o feature selection.

_
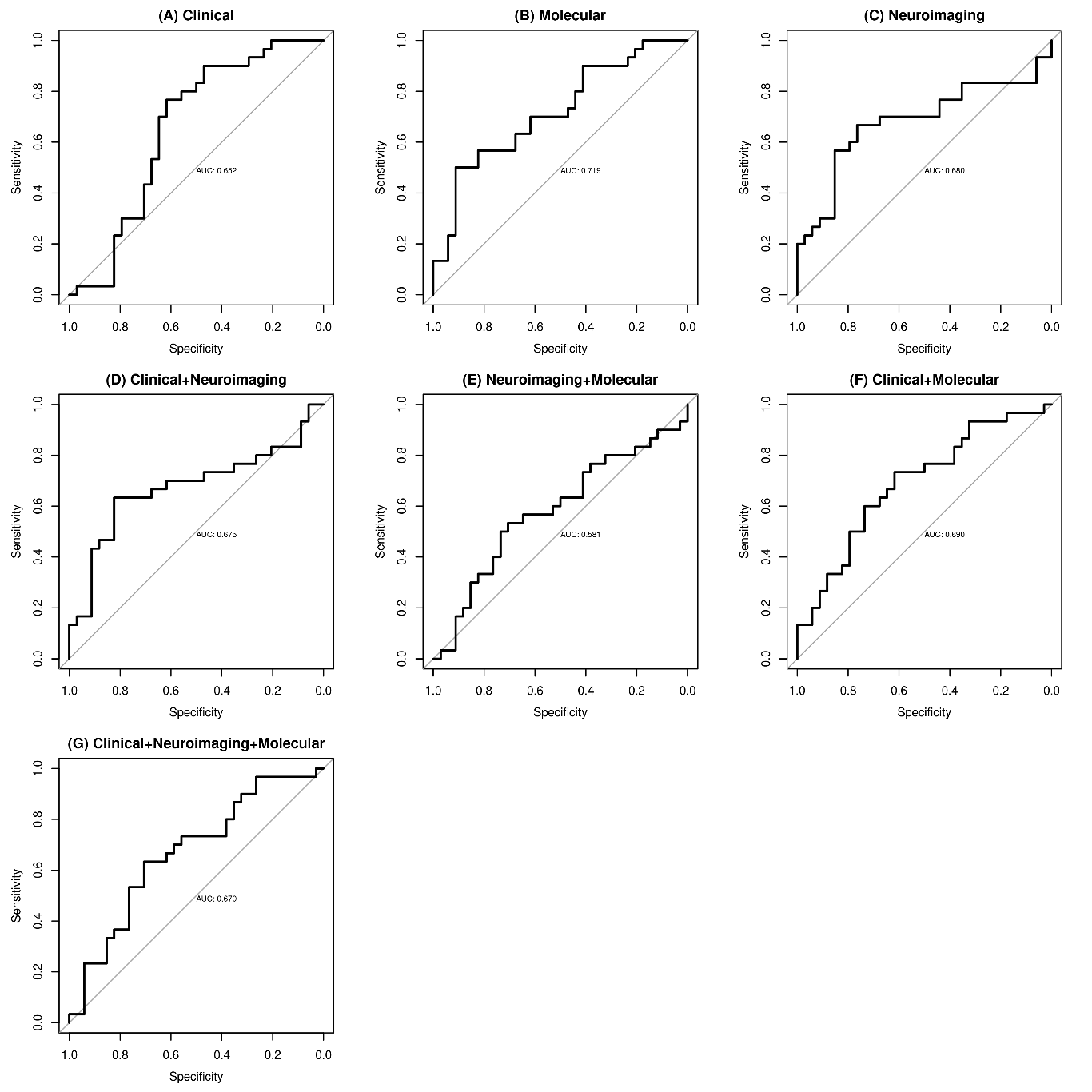
_

**Figure S8:** **Operating Characteristic (ROC) curves for tier 2 week 0 predictor models.** The most predictive (highest AUC) model was chosen for each predictor modality and combination: (A) clinical data, random forest model with feature selection (B) molecular data, elastic net model w/o feature selection (C) neuroimaging data, GBM model w/o feature selection (D) clinical + neuroimaging, random forest with feature selection (E) neuroimaging + molecular, SVM model with feature selection (F) clinical + molecular, Naive Bayes model with feature selection (G) clinical + neuroimaging + molecular, SVM model with feature selection.


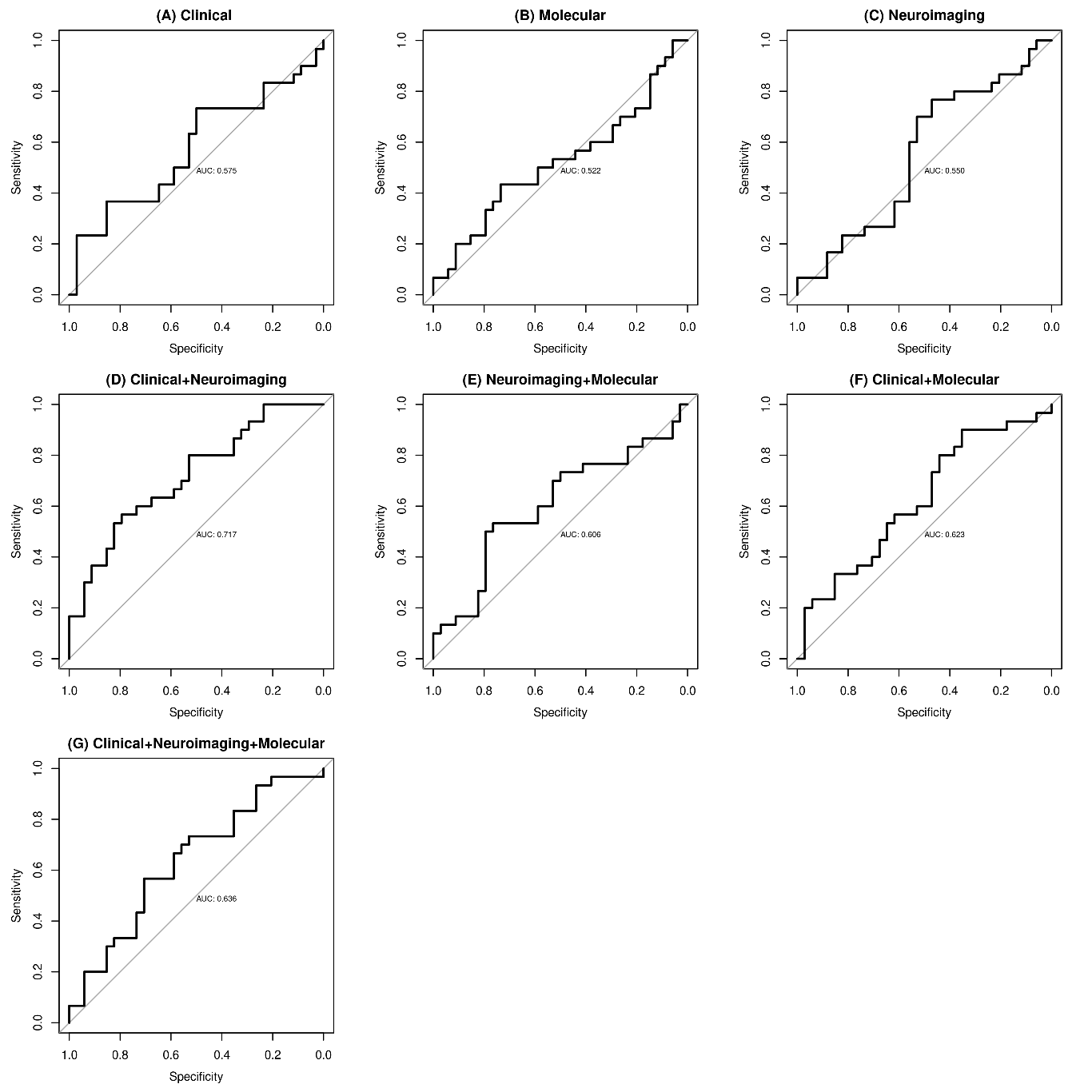


**Figure S9:** **Receiver Operating Characteristic (ROC) curves for tier 1 week 0 + week 2 predictor models.** The most predictive (highest AUC) model was chosen for each predictor modality and combination: (A) clinical data, elastic net model w/o feature selection (B) molecular data, SVM model w/o feature selection (C) neuroimaging data, Naive Bayes model w/o feature selection (D) clinical + neuroimaging, random forest w/o feature selection (E) neuroimaging + molecular, Naive Bayes model w/o feature selection (F) clinical + molecular, elastic net model w/o feature selection (G) clinical + neuroimaging + molecular, random forest model w/o feature selection.


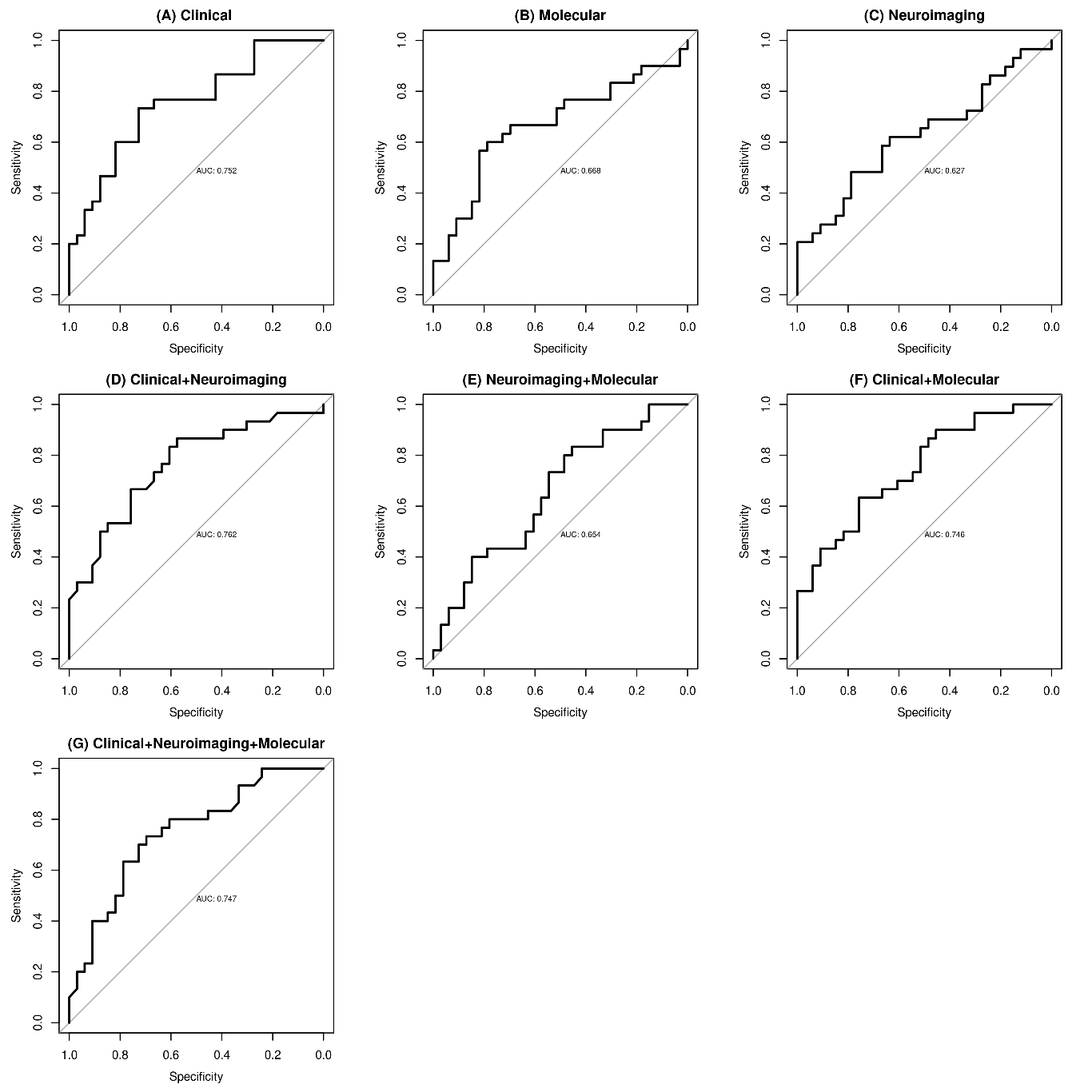

Supplement: Supplementary file 1 [file S0033291722002124sup001.docx]
